# Closely related, yet unique: Distinct homo- and heterodimerization patterns of G protein coupled chemokine receptors and their fine-tuning by cholesterol

**DOI:** 10.1371/journal.pcbi.1006062

**Published:** 2018-03-12

**Authors:** Stefan Gahbauer, Kristyna Pluhackova, Rainer A. Böckmann

**Affiliations:** Computational Biology, Department of Biology, Friedrich-Alexander University Erlangen-Nürnberg, Erlangen, Germany; University of Virginia, UNITED STATES

## Abstract

Chemokine receptors, a subclass of G protein coupled receptors (GPCRs), play essential roles in the human immune system, they are involved in cancer metastasis as well as in HIV-infection. A plethora of studies show that homo- and heterodimers or even higher order oligomers of the chemokine receptors CXCR4, CCR5, and CCR2 modulate receptor function. In addition, membrane cholesterol affects chemokine receptor activity. However, structural information about homo- and heterodimers formed by chemokine receptors and their interplay with cholesterol is limited. Here, we report homo- and heterodimer configurations of the chemokine receptors CXCR4, CCR5, and CCR2 at atomistic detail, as obtained from thousands of molecular dynamics simulations. The observed homodimerization patterns were similar for the closely related CC chemokine receptors, yet they differed significantly between the CC receptors and CXCR4. Despite their high sequence identity, cholesterol modulated the CC homodimer interfaces in a subtype-specific manner. Chemokine receptor heterodimers display distinct dimerization patterns for CXCR4/CCR5 and CXCR4/CCR2. Furthermore, associations between CXCR4 and CCR5 reveal an increased cholesterol-sensitivity as compared to CXCR4/CCR2 heterodimerization patterns. This work provides a first comprehensive structural overview over the complex interaction network between chemokine receptors and indicates how heterodimerization and the interaction with the membrane environment diversifies the function of closely related GPCRs.

## Author summary

G protein coupled chemokine receptors are proteins embedded in the cell membrane. They play essential roles in the human immune system. Moreover, chemokine receptors are also involved in various diseases including cancer metastasis and HIV infection leading to AIDS. In case of the chemokine receptors CXCR4, CCR5, and CCR2, recent studies revealed that the proteins associate to so-called dimers, consisting of two receptors. This dimerization was shown to regulate protein function. Consequently, the association of chemokine receptors increasingly gained attention for modern drug design. However, structural information about chemokine receptor dimers is scarce due to experimental limitations. Here, we present first atomistic insight into chemokine receptor dimer structures consisting of either two equal receptors (homodimers) or of two different receptors (heterodimers). To this end, we employed thousands of molecular dynamics simulations of the receptor association process. The simulations revealed similar homodimerization patterns for the closely related receptors CCR5 and CCR2, and pinpointed the dimerization hotspots on both proteins. Cholesterol was found to differentiate the association patterns between CXCR4 and the related CC chemokine receptors, indicating that both heterodimerization and the interplay with cholesterol play important roles in fine-tuning chemokine-related signaling pathways.

## Introduction

G protein coupled receptors (GPCRs) constitute one of the largest groups of cell surface transmembrane receptors [1]. All members of this protein family share the typical architecture of seven transmembrane helices (TM1-7) connected via three intra- and three extracellular loops (ICL and ECL, respectively). In general, the receptors sense external signals with their extracellular segment, while G proteins couple to the intracellular domain. Usually, an additional amphipathic helix 8 (H8) is formed by residues between the last transmembrane helix (TM7) and the intracellular C-terminus [2].

A small subgroup of class A GPCRs is formed by so-called chemokine receptors [3]. These receptors cater for the targeted migration of cells belonging to the immune system and play an essential role in the inflammatory response [4]. Consequently, chemokine receptors became intriguing candidates for therapeutic targets in inflammatory diseases [5]. In addition, certain chemokine receptors have been reported to play important roles in tumor progression as well as metastasis [6] and were shown to function as co-receptors for the human immunodeficiency virus (HIV) during cell infection [7]. Depending on the relative positions of the N-terminal cysteine residues of the agonist chemokine, typical chemokine receptors can be divided into four subfamilies: XCR1, CCR(1-10), CXCR(1-6), and CX_3_CR1 [8].

As reported for a continuously growing number of GPCRs [9], several chemokine receptors have been indicated to function as homo- or heterodimers or even oligomers of higher order [10–12]. The assembly of GPCRs was indicated to have important functional consequences, it may e.g. affect the cooperativity between ligand binding sites, alter the intracellular transport of GPCRs, or activate different signaling pathways [13–16]. For class A GPCRs, the interactions between receptors are mainly formed by their transmembrane helices. Consequently, certain membrane components, e.g. cholesterol or polyunsaturated lipids, have been reported to modulate the function and association of GPCRs [17–22]. Notably, cholesterol is frequently required in the crystallization process of GPCRs and several X-ray crystal structures show cholesterol molecules bound between adjacent protomers under crystal conditions [21, 23–26].

Especially three chemokine receptors, namely CCR2, CCR5, and CXCR4 have been shown repeatedly to homo- and heterodimerize and the association was indicated to modulate receptor function [27–48]. Homo- and heterodimerization were reported as either ligand-promoted [49–51] or constitutive [35, 52, 53]. This discrepancy could be coupled to interpretational difficulties since differences in the measured dimerization data—e.g. from Förster or bioluminescence resonance energy transfer studies [54] (FRET or BRET, respectively)—could result from conformational changes in preexisting dimers upon ligand treatment instead of alterations of the binding affinity between receptors [14, 21, 35]. Furthermore, the modulation of chemokine receptor function by membrane cholesterol has been reported in several experimental studies [53, 55–63].

We recently studied the spontaneous assembly of CXCR4 receptors in different membrane compositions [47] using ensembles of molecular dynamics (MD) simulations, in order to unravel the effects of cholesterol on the receptor dimerization at atomistic resolution. Our results revealed that cholesterol affects the homodimerization via binding between TM1 and TM7, thus preventing these helices from engaging in dimer interactions as observed in cholesterol-free membranes. In turn, the intercalation of cholesterol between monomers induced a symmetric interface including TM3 and TM4 of both receptors (TM3,4/TM3,4 dimer) [47]. Interestingly, peptides derived from TM4 of CXCR4 were shown before to affect the dimerization and further to strongly weaken the activity of the receptor [35, 53].

The homodimerization of CCR5 was shown in various studies and suggested to impair HIV infection [28]. CCL5 (RANTES), an agonist chemokine of CCR5, was suggested to stabilize receptor dimers [28, 32], however, constitutive agonist-independent assembly of CCR5 was reported as well [31, 32, 37]. In addition, monoclonal antibodies (mAb) were constructed for CCR5 that can stabilize homodimers [28, 30]. Ile52 on TM1 and Val150 on TM4 have been proposed as crucial for both, CCR5 dimerization and signaling, since double mutants of the receptor showed neither dimerization nor function [32]. However, these findings were challenged by other experiments [64]. The chemokine receptor CCR2 is closely related to CCR5, the receptors share ≈ 75% overall sequence identity and their transmembrane segments show up to 92% identity (see S1 Fig) [38]. As reported for CXCR4 and CCR5, CCR2 was shown to homodimerize [29, 46] and ligand stimulated receptors altered the dimer configuration [35]. Furthermore, similar to CCR5, residues Val64 on TM1 and Ile164 (according to Uniprot—ID: P41597—Ile163) on TM4 were reported to be involved in CCR2 dimerization since double mutants were unable to trigger signaling [32]. In addition, a heptapeptide containing the residues Met61—Leu67 of the TM1 of CCR2 was shown to inhibit CCR2-mediated cell migration by impeding the receptor homodimerization [65].

Besides homodimerization, heterodimerization of CXCR4, CCR5, and CCR2 was observed in numerous experiments and could be connected to modulations of ligand binding and receptor function [12, 44]. The constitutive heterodimerization or -oligomerization of these chemokine receptors was reported in several studies [36, 38, 40, 42, 66] and ligand-binding to the receptor complexes was described to induce conformational changes in heterodimers [35, 40, 42, 46]. Negative ligand-binding cooperativity of allosteric nature across chemokine receptor heteromers was reported, suggesting that usually only one ligand binds to a complex of receptors which induces conformational changes at the ligand-binding site of the associated receptor [12, 38, 39, 41]. Additionally, heteromerization was reported to enable chemokine receptors to couple to different kinds of G proteins as compared to receptor homomers and monomers [12, 29, 38, 41, 46, 67].

Especially the assembly of CXCR4 and CCR5 gained a great deal of attention since both receptors can act as co-receptors for HIV. It was shown that each receptor can associate with the transmembrane glycoprotein cluster determinant 4 (CD4) and that these associates are used by HIV to enter the cell. On the other hand, CD4/CXCR4/CCR5 heterotrimers revealed negligible binding to the virus envelope [45]. In addition, a mutant of CCR2 (Val64Ile) was revealed to delay the development of AIDS in HIV-infected individuals potentially due to heterodimerizing with CXCR4 or CCR5 [68]. However, more recent BRET studies could not indentify different heterodimerization affinites between CXCR4 and wildtype CCR2 or the CCR2 Val64Ile variant [35]. Furthermore, inducing the oligomerization of CCR2 with CCR5 and CXCR4 with a specific monoclonal antibody was reported to block HIV-entry [34]. In addition, the depletion of cholesterol from cellular membranes was reported to reduce the ability of HIV to fuse with host cells, likely by affecting the clustering of coreceptors at cell membranes [57, 60].

Based on the impact of GPCR dimerization on receptor function, novel therapies are developed to target protein association [13]. Especially the construction of bivalent ligands that are able to bind both receptors in homo- or heterodimeric GPCR complexes have been proposed as potent targets to regulate GPCR function [69–71]. However, the rational design of new drugs targeting GPCR dimers is subject to the availability of receptor complex structures [72]. So far, structural information regarding GPCR homodimer interfaces is most frequently derived from available crystal structures [2].

During the last years, more and more human class A GPCRs have been solved with X-ray crystallography and different dimeric contacts could be observed [21, 47]. Thereby, different receptors revealed distinct homodimer interfaces and the aggregation of receptors is likely dependent on the crystallization conditions [73–75]. In addition, certain receptors (e.g. the *β*_2_-adrenergic receptor) could be crystallized in different dimeric configurations, indicating a promiscuous association of GPCRs [23, 76].

The structures of the chemokine receptors CXCR4, CCR5, and CCR2 have also been determined with X-ray crystallography during the last years [77–80]. CXCR4 bound to either a small antagonist (IT1t), a cyclic antagonist (CVX15), or a viral antagonist chemokine (vMIP-II) revealed a symmetric TM5,6/TM5,6 dimer interface as the dominant interaction mode under crystallization conditions [77, 78]. However, the crystal packing also revealed TM1/TM5-7 and TM1/TM1 contacts [47]. CCR5 in complex with the HIV drug maraviroc showed dimer contacts between TM1,7 and H8 of one monomer and TM4 and TM5 of an adjacent monomer [79]. Interestingly, the previously described residues Ile52 (TM1) and Val150 (TM4) that were suggested to be crucial for CCR5 dimerization and signaling [32] are located on the contacting helices, however, neither residue is directly involved in the crystallographic dimer interface. Recently, the crystal structure of CCR2 in complex with an orthosteric (BMS-681) and allosteric (CCR2-RA-[R]) antagonist was determined [80]. Here, no physiological relevant dimer interface could be observed since the protomers are exclusively oriented antiparallel in the crystal packing. In addition, the chemokine receptor CCR9 revealed a symmetric TM4/TM4 interface under crystallization conditions [81].

Thus far, no crystallographic data on GPCR heterodimers is available due to the enormous complexity of the crystallization process. However, *in silico* methods such as molecular dynamics (MD) simulations have proven useful to provide molecular information about the dimerization of transmembrane proteins [82–85]. Especially coarse-grained (CG) simulations are a commonly used method to study GPCR assembly, since they allow to explore biological systems on the micro- to millisecond timescale [47, 82, 86–93]. Dimer and oligomer conformations of several GPCRs could be determined and the influence of membrane properties such as bilayer thickness [86, 87] or the presence of specific membrane components were investigated [90]. The interactions between certain lipid types [90, 94–97] or cholesterol and GPCRs were also studied in CG and atomistic simulations and specific cholesterol binding spots on GPCR monomers and dimers have been found for several receptors [47, 96, 98–102]. Besides the spontaneous association of GPCRs, the binding strength of different dimer interfaces can be approximated with CGMD simulations [103–108]. From these studies, energetically favored dimer interfaces and possible energy barriers for dimer formations resulting from trapping of lipids at the interfaces could be elucidated [91, 105].

In this work, the spontaneous homo- and heterodimerization of the chemokine receptors CXCR4, CCR5, and CCR2 is investigated using ensembles of molecular dynamics simulations. Our results reveal that the closely related CC chemokine receptors CCR5 and CCR2 share common features with regard to dimerization and interactions with cholesterol that differ from the previously reported characteristics of CXCR4 [47]. In addition, we provide first structural information about chemokine receptor heterodimers and elucidate the role of cholesterol in differentiating between the heterodimerization of CXCR4 and the CC chemokine receptors.

## Results

The homo- and heterodimerization of the chemokine receptors CXCR4, CCR5, and CCR2 was analyzed from ensembles of corresponding dimerization MD simulations [82] at coarse-grained resolution. In detail, each chemokine receptor system was studied in 500 independent self-association simulations on the microsecond timescale. In each simulation, two randomly rotated receptors were placed at an initial minimal distance of ≈ 3.5 nm into either a pure palmitoyl-2-oleoyl-sn-glycero-3-phosphocholine (POPC) bilayer or a POPC bilayer with 30 mol% cholesterol content (see Table 1 for a summary of the different setups). This approach allows for an unbiased determination of dimerization interfaces of transmembrane peptides or proteins [47, 82, 83]. Obtained simulation ensembles were analyzed for self-association rates, preferred dimerization interfaces and the influence of cholesterol on dimerization, as well as specific cholesterol binding sites.

**Table 1:** Summary of dimerization simulation setups.

Receptors	Membrane	Simulation time ^a^	Number of simulations ^b^	Number of dimers ^c^	*k* ^d^ in 10^6^ *s*^−1^	$\Delta G_{lower-bound}^{cg}$ ^e^ in kJ/mol
**CXCR4/CXCR4** ^f^	POPC	3 *μs*	501	251	0.274	-24.95
	POPC/30% chol	6 *μs*	499	129	0.057	-19.84
**CCR5/CCR5**	POPC	3 *μs*	506	196	0.190	-18.95
	POPC/30% chol	8 *μs*	503	190	0.063	-17.19
**CCR2/CCR2**	POPC	3 *μs*	507	249	0.239	-19.61
	POPC/30% chol	8 *μs*	498	212	0.079	-18.71
**CXCR4/CCR5**	POPC	3 *μs*	505	274	0.281	-19.92
	POPC/30% chol	8 *μs*	511	244	0.084	-19.27
**CXCR4/CCR2**	POPC	3 *μs*	505	275	0.302	-20.48
	POPC/30% chol	8 *μs*	503	209	0.075	-18.79
**CCR5/CCR2**	POPC	3 *μs*	501	216	0.224	-18.84
	POPC/30% chol	8 *μs*	499	207	0.074	-18.17

*^a^* Simulation time of each simulation.

*^b^* Number of performed simulations.

*^c^* Number of dimers at the end of the simulation time.

*^d^* First order reaction rates derived from S2b Fig. The area density of receptors in the simulation setups was ≈ 0.015nm^−2^.

*^e^* Lower bound estimates for the binding free energies calculated from coarse-grained (cg) simulations (see Materials and methods, and S2c Fig).

*^f^* The data of the homodimerization of CXCR4 was taken from our previous study [47] (included for comparison).

### Chemokine receptor-specific influence of cholesterol on homo- and heterodimerization rates

**Fig 1:**
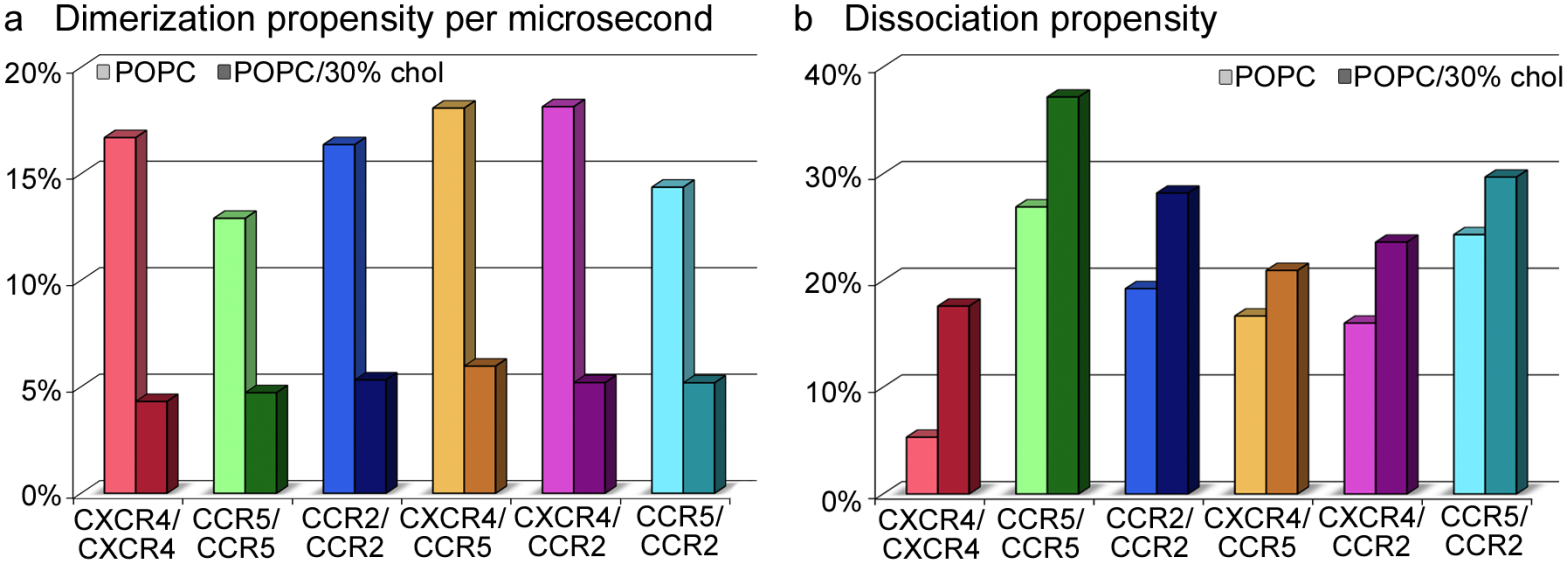
Dimerization and dissociation propensities of the studied chemokine receptors. **a** The dimerization propensity is given as the relative number of dimers formed per microsecond and was calculated as the ratio between the final number of dimers divided by the total simulation time in microseconds. **b** Dissociation propensities were computed by dividing the total number of dissociation events (see S2d Fig) by the total number of dimerization events for most populated dimer interfaces (see Materials and methods).

In the coarse-grained simulation ensembles, receptors were considered as dimers if the interaction energy (calculated as the sum of the Lennard-Jones and Coumbic interaction energies) between the transmembrane segments reached -50 kJ/mol, indicating direct protein-protein contacts (see Materials and methods Fig 8). Chemokine receptor dimerization was observed at rates of 0.2–0.3 *μs*^−1^ in pure POPC and 0.05–0.08 *μs*^−1^ in POPC/30% cholesterol membranes. A comparable number of dimers for these different lipid environments was obtained for simulation lengths of 3 *μs* and 8 *μs*, respectively (compare Fig 1 and Table 1). Interestingly, the homo- and heterodimerization between CC chemokine receptors in POPC revealed lower rates as compared to the homo- and heterodimerization involving CXCR4. The strongest effect of cholesterol on the dimerization rate was observed for the homodimerization of CXCR4, that was reduced by 80% in presence of cholesterol.

Lower bound estimates for the binding free energy between chemokine receptors in coarse-grained simulations could be derived from the ratio between the total simulation time spent in monomeric states following the dissociation of a spontaneously assembled dimer complex, and the total time the system spent in dimeric states (see Materials and methods, S2c Fig and [47]). The binding free energies were computed in a range from -25 to -17 kJ/mol for the different chemokine receptor dimers (see Table 1). As depicted in Fig 1b and S2d Fig, the dissociation was significantly enhanced for the homo- and heterodimerization involving CC chemokine receptors in pure POPC membranes as compared to the homodimerization of CXCR4 [47] (by a factor of 3–5).

Presence of cholesterol increased the dissociation propensity for chemokine receptor homo- and heterodimers. Interestingly, this clear trend was mitigated for compact dimer interfaces (i.e. for higher interaction energies between the receptor transmembrane segments), indicating that cholesterol complicates the formation of compact dimers from inital protein-protein contacts (see S3 Fig). Especially, the dissociation propensity for the homodimerization of CXCR4 was increased by a factor of ≈ 3, resulting in a pronounced decrease in binding affinity by 4 kJ/mol in cholesterol-rich membranes, while the homo- and heterodimerization of all other receptor combinations were less affected.

30% cholesterol content decreased the receptor diffusion by ≈ 50% as compared to the diffusion in pure POPC (see S1 Table). In contrast, the dimerization rates were decelerated by approx. 70-80% in cholesterol-rich lipid bilayers. Thus, assuming a simple hit-and-dimerize model, cholesterol affected the dimerization of chemokine receptors not only by slowing down the protein self-diffusion, but as well by modulating the protein association process [47]. Therefore, specific binding of cholesterol to monomeric chemokine receptors is analyzed in detail in the following Section.

### Cholesterol-binding to suggested dimerization sites

Based on the observation that cholesterol modulates the association of chemokine receptors, interactions between the studied GPCRs and the sterol were analyzed based on simulations of the different receptors in monomeric form in membranes at 10% cholesterol content.

**Fig 2:**
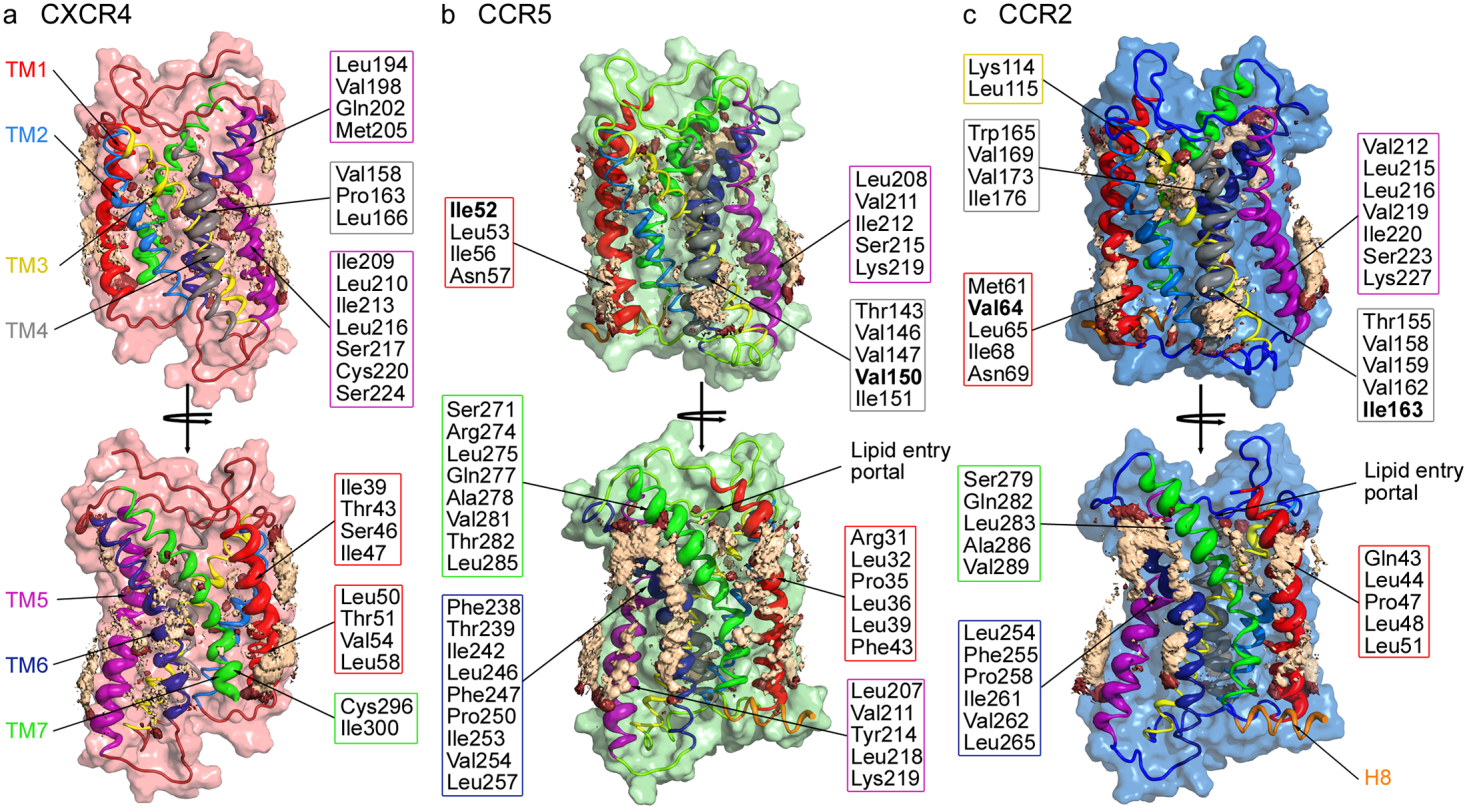
Cholesterol binding to CXCR4, CCR5, and CCR2 chemokine receptor monomers. The spatial distributions of the five closest cholesterol molecules around the chemokine receptors (**a**) CXCR4, (**b**) CCR5, and (**c**) CCR2, are shown in light orange, the spatial distributions of the polar headgroup of cholesterol (ROH beads) are shown in dark red. Receptor helices are colored according to the following scheme: TM1: red, TM2: light blue, TM3: yellow, TM4: grey, TM5: purple, TM6: dark blue, TM7: green, H8: orange. The helix thickness encodes the residue-resolved cholesterol-occupancy. Residues showing high cholesterol occupancies are explicitely listed and enframed according to the transmembrane helix they are located on. Residues previously identified in experiments as important for chemokine receptor function [32, 68] are highlighted in bold. The data for CXCR4 (**a**) was taken from our previous study [47] and included for comparison.

Specific binding sites are highlighted by the spatial distribution densities of cholesterol around the receptor combined with the contact time of helix residues with cholesterol (Fig 2). As described previously [47], cholesterol strongly bound to CXCR4 between the TM1 (*red*) and TM7 (*green*) helices close to the intracellular water-membrane interface, covered the extracellular half of TM1, and padded the rugged surface on TM5 (see Fig 2a, TM5 in *purple*).

Interestingly, the cholesterol binding patterns on the closely related CC chemokine receptors largely coincided but differed substantially from CXCR4. The common binding spots on the CC chemokine receptors included regions between the extracellular-facing halves of TM6 and TM7, intracellular-facing residues on TM5, both intra- and extracellular halves of TM1 and the intracellular part of TM4 (see Fig 2b and 2c). For both CC chemokine receptors it was observed that cholesterol molecules reached with their polar headgroups into a lipid entry portal between the extracellular halves of TM1 and TM7. Furthermore, each CC chemokine receptor revealed an additional cholesterol binding spot albeit with lower density: In detail, on CCR5 an additional cholesterol molecule occupied the cytosolic half of TM5 and CCR2 bound an extra cholesterol between TM3 and TM4.

In case of CCR5, the intracellular-facing binding spot on TM1 includes the previously discussed Ile52, indicated to play an important role in receptor function [32]. Similarly, in the corresponding binding site on CCR2, the experimentally addressed Val64 [32, 68] showed a (moderate) cholesterol occupancy. Interestingly, the residues Met61—Leu67, used for the design of CCR2 homodimerization blocking heptapeptides [65], construct the cholesterol binding spot at the intracellular half of CCR2. Additionally, the residues Val150 (CCR5) or Ile163 (CCR2), recognized as important for both function and dimerization [32], contributed to the cholesterol binding spot on TM4. How binding of cholesterol to chemokine receptors modulates CC chemokine receptor homodimer interfaces is discussed in the following Section.

### CC chemokine receptors share similar homodimerization patterns distinct from CXCR4

Chemokine receptor dimerization patterns were analyzed from the relative orientations of the receptor monomers in spontaneously formed dimer configurations (described in detail in [47], see also Materials and methods, and Supplementary Information).

**Fig 3:**
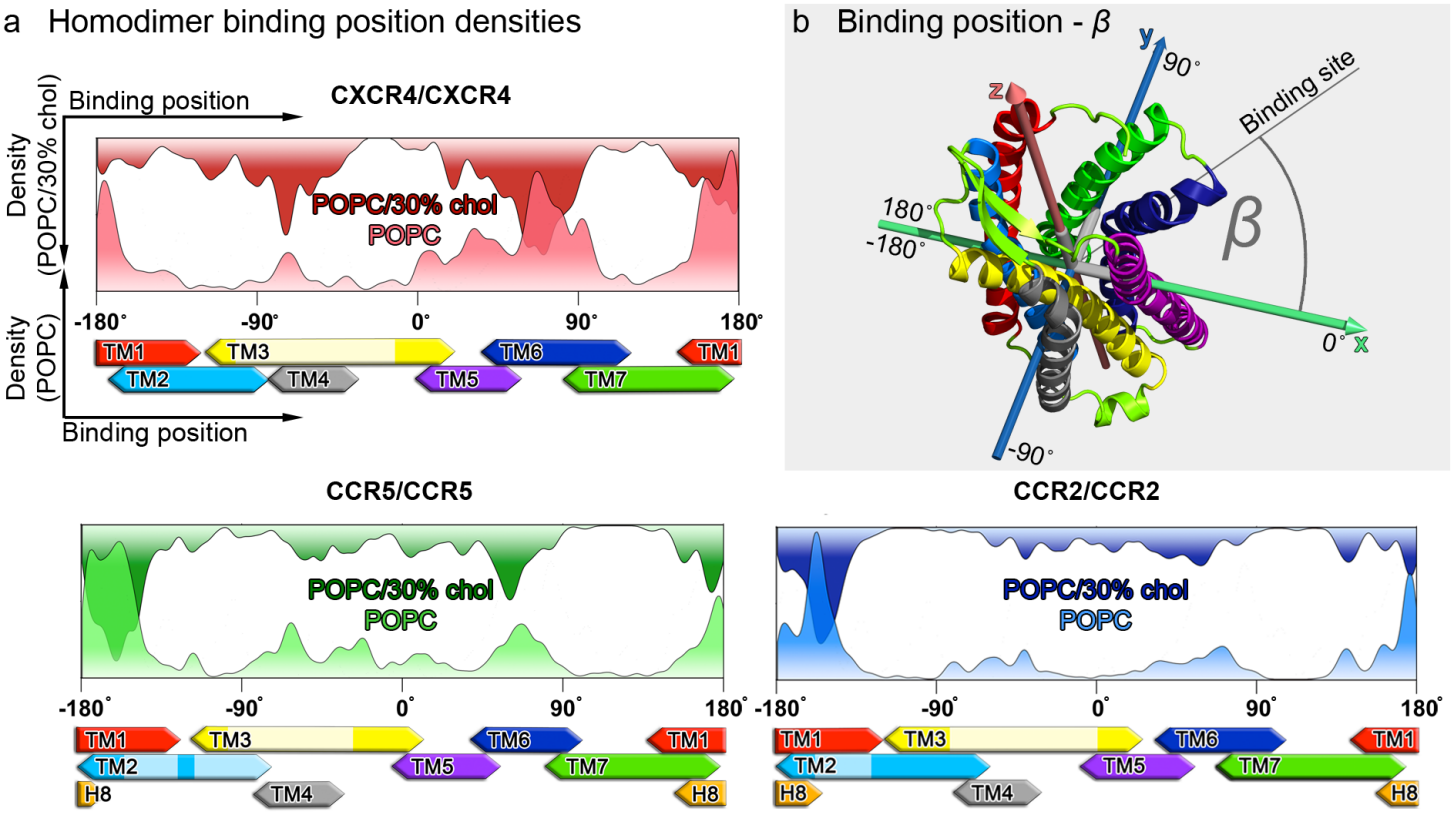
Involvement of individual transmembrane helices at homodimer interfaces. **a** Homodimer binding position densities (see Materials and methods), i.e. involvement of individual transmembrane helices at chemokine receptor homodimer interfaces obtained after 3 *μs* in pure POPC membranes (bottom-up), and after 8 *μs* (6 *μs* in case of CXCR4) in POPC membranes containing 30% cholesterol (top-down). The data for CXCR4 were taken from our previous study [47]. Binding position angles are assigned to individual transmembrane helices according to **b** (sample coordinate system based on the principal axes of CCR5). Transmembrane helices are colored according to the scheme established in Fig 2. Bleached transmembrane segments (on TM2 and TM3) at the x-axis label correspond to TM areas that are not exposed at the protein surface.

Relative binding angles between protomers at dimer interfaces can be assigned to individual transmembrane helices (see Fig 3), thus allowing to compute the relative involvement of helices at dimer interfaces. The closely related CC chemokine receptors shared very similar homodimerization patterns (see Fig 3a), likely due to the high sequence identity within their transmembrane domains (see S1 Fig). As shown in Fig 3, while TM1, TM5,6 and 7 of CXCR4 displayed equally high binding densities in homodimers in pure POPC, the presence of cholesterol reduced the involvement of TM1 but increased the binding density on TM4 [47]. In case of CC chemokine receptors, especially TM1 was revealed as a homodimerization hotspot. This observation is in good agreement with previous experimental studies, where mutations on TM1 or heptapeptides derived from the sequence of TM1 hampered CC chemokine receptor homodimerization [32, 65]. Furthermore, the presence of cholesterol did not affect the binding position densities of CC chemokine receptors as strongly as previously reported for CXCR4 [47].

**Fig 4:**
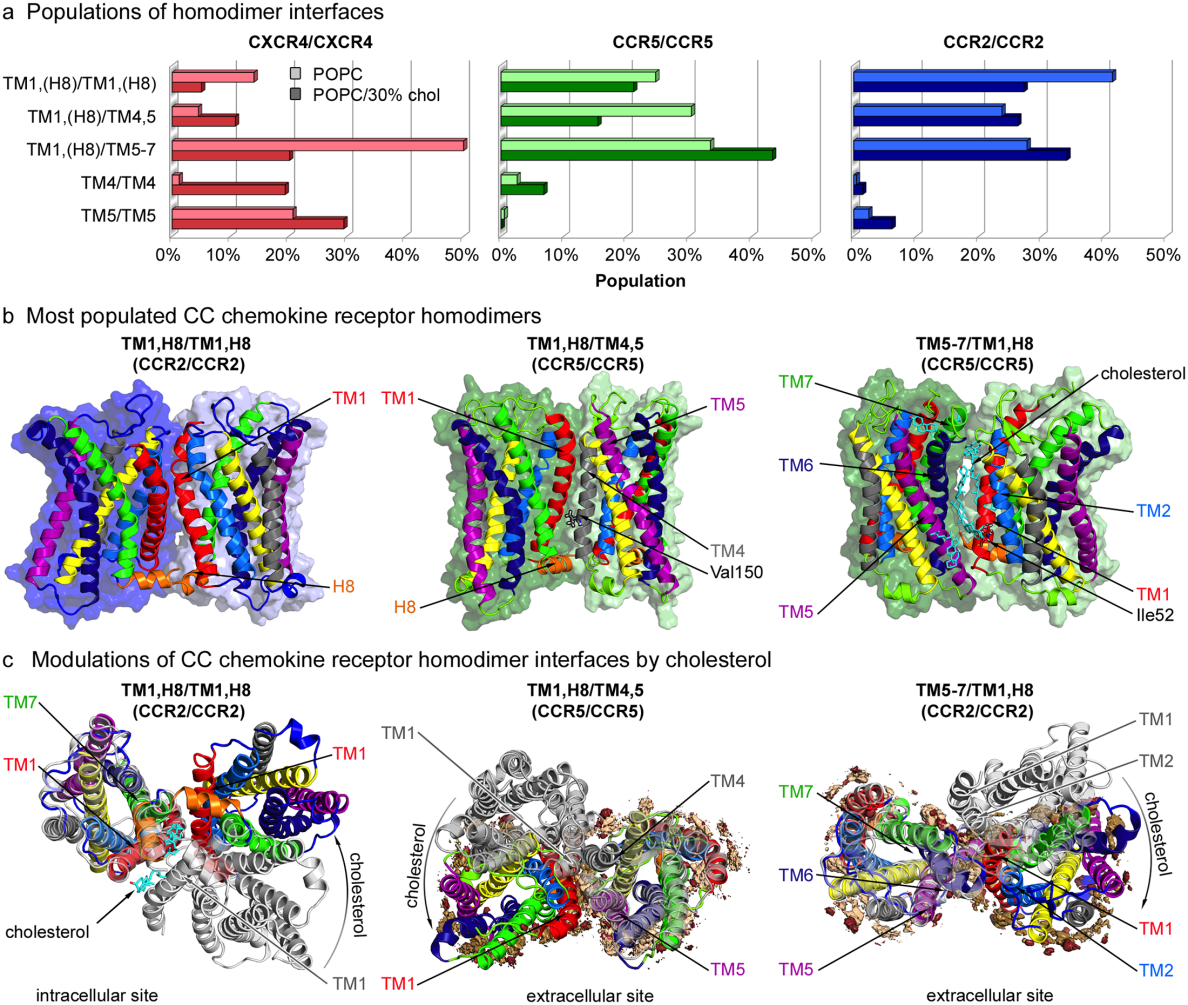
Chemokine receptor homodimer interfaces. **a** Populations of the five most populated homodimer interfaces after 3 *μs* in pure POPC membranes (light colors), and after 8 *μs* (6 *μs* in case of CXCR4) in POPC membranes containing 30% cholesterol (dark colors). The data for CXCR4 were taken and generalized from our previous study [47]. **b** Side views on the representative CC chemokine receptor homodimer structures for the most populated interfaces (see Materials and methods). Structures are colored consistent with Fig 2. Individual cholesterol molecules are shown in cyan. **c** Effect of cholesterol on the dimerization interfaces. Spatial distributions of the five nearest cholesterol molecules around each monomer are shown in light and dark orange whereas the spatial distributions of the molecules polar head groups (ROH beads) are shown in dark red. Cholesterol-free dimers are shown in light grey, whereas cholesterol-containing structures follow the color scheme of Fig 2.

The homodimer populations of CCR2 and CCR5 are distinct from dimer configurations reported previously for CXCR4 [47] (see Fig 4a). Overall, three main homodimer interfaces could be observed for the CC chemokine receptors: TM1,H8/TM1,H8, TM1,H8/TM4,5 and TM1,H8/TM5-7 (see Fig 4b). In case of CXCR4, the interface formed by interacting TM1 and TM5-7 helices was most abundant in cholesterol-free membranes, whereas the TM1/TM1 and TM1/TM4,5 motifs that were strongly populated for CC chemokine receptors did not contribute significantly to the homodimerization pattern of CXCR4. Oppositely, symmetric TM4/TM4 or TM5/TM5 dimer interfaces as observed for CXCR4 were barely obtained for CC chemokine receptors (see S4a Fig for representative structures of less populated CC chemokine receptor homodimers). Notably, H8 contributed to the main CC chemokine receptor homodimer interfaces. However, helix 8 was not resolved in any currently available crystal structure of CXCR4 [77, 78]. Especially in pure POPC membranes, CCR5 and CCR2 homodimers showed very similar configurations (see relative binding angles of monomers in homodimers in S5b and S5c Fig).

Due to the observation, that the Martini coarse-grained model may overestimate the aggregation between proteins [109], we cannot exclude artificial dimer configurations among the less populated dimer interfaces. However, as also shown in previous studies [47, 83], highly populated dimer configurations observed in simulation ensembles compare well to experimental findings.

The CCR5 TM1,H8/TM4,5 dimer configuration deviates by 6.7 Å (backbone RMSD of transmembrane region) from the dimer configuration observed in the CCR5 crystal structure [79]. Under crystallization conditions, the proteins in the complex are aligned parallel in periodic chains, while the spontaneously formed TM1/TM4,5 dimer interface derived from simulations adopted an overall more compact configuration (see S6 Fig). The binding position between the monomers of the corresponding self-assembled dimer was shifted by 35° as compared to the crystal complex, allowing for significantly more contacts between the TM1 and TM4 helices at the interface and an accordingly increased buried surface area at the interface of 22.5nm^2^ compared to only 14.7nm^2^ for the crystal complex. In addition, the above described CC homodimer configurations are indirectly supported by a mutation study: Val150 of CCR5 and Ile163 of CCR2 (both on TM4) were suggested as important for homodimerization and function of the respective receptors [32]. Both residues are centrally located at the dimerization interface of the self-assembled TM1,H8/TM4,5 dimer (see Fig 4b). In turn, Val150 barely contributes to the crystal dimer interface of CCR5 (see S6b Fig).

#### The homodimerization response to cholesterol differs between the closely related CC chemokine receptors

The presence of cholesterol did not affect the populations of CC chemokine receptor homodimer interfaces as strongly as it was observed for CXCR4 [47]. However, the dimerization response to cholesterol differed between these homologous receptors: Cholesterol showed a stronger impact on both the population and conformation of the CCR2 TM1,H8/TM1,H8 dimer as compared to the corresponding CCR5 dimer. As displayed in Fig 4c, cholesterol binding to the intracellular halves of TM1 of CCR2 broke the symmetry due to spatial hindrance of TM1/TM1 contacts formed in cholesterol-free POPC membranes. Instead, the binding site of the cholesterol-containing CCR2 dimer was shifted by ≈ 40° (see Fig 4c and S5a3 Fig) and mostly involved interactions between the extracellular halves of TM1 and additional contacts between TM1 and TM7 of the interacting receptors. At this interface, a cholesterol molecule could bind between TM1 and TM7 of one receptor. Overall, the presence of cholesterol decreased the amount of CCR2 TM1,H8/TM1,H8 homodimers.

The CC chemokine receptor TM1,H8/TM4,5 homodimer underwent a comparably smaller conformational adjustment, shifting the relative binding angle by ≈ 29° (see Fig 4c and S5a2 Fig) resulting in an enhanced contribution of TM5 to the dimer interface (see Fig 4c). Even though both CC chemokine receptors revealed a similar conformational change at this dimer interface in response to cholesterol, only the population of CCR5 TM1,H8/TM4,5 dimers was reduced in cholesterol-containing membranes.

As shown in Fig 4b, cholesterol molecules could intercalate between the receptors in the TM1,H8/TM5-7 homodimer configuration. The typical binding pattern involved the cholesterol binding sites on TM5, TM6, and TM7 of one receptor and TM1 of the interaction partner (compare Fig 2). Interestingly, intercalating cholesterol molecules at this dimer interface also bound to Ile52, a residue shown to be important for CCR5 dimerization and function [32]. In case of CCR2, binding of cholesterol to the main cholesterol-binding spot between TM6 and TM7 hindered both helices to interact with the other receptor and the dimerization position was shifted by roughly 15° to include more contacts between TM1 and TM5 at the interface (see Fig 4c, right panel). Both CC chemokine receptors showed increased populations of TM1,H8/TM5-7 interfaces in cholesterol-containing membranes. In contrast, the population of TM1/TM5-7 homodimers of CXCR4 was strongly reduced in presence of cholesterol [47] likely resulting from the strong and almost complete cholesterol occupation of TM1 of CXCR4 (see Fig 2a), while the CC chemokine receptors showed weaker, only partial occupations of TM1 (see Fig 2b and 2c).

The different effects of cholesterol on the homodimerization of the two studied CC chemokine receptors discussed above elucidate a complex interaction network between closely related receptors that is fine-tuned by membrane cholesterol.

### Type-specific heterodimerization characteristics of chemokine receptors

Apart from homodimerization of chemokine receptors, also heterodimerization between CXCR4, CCR5, and CCR2 was observed in experiments and their importance for function discussed [48]. Here, we report the first structural data for chemokine receptor heterodimers obtained from CG simulations.

#### CCR5 and CCR2 reveal distinct, cholesterol-sensitive dimerization patterns with CXCR4

**Fig 5:**
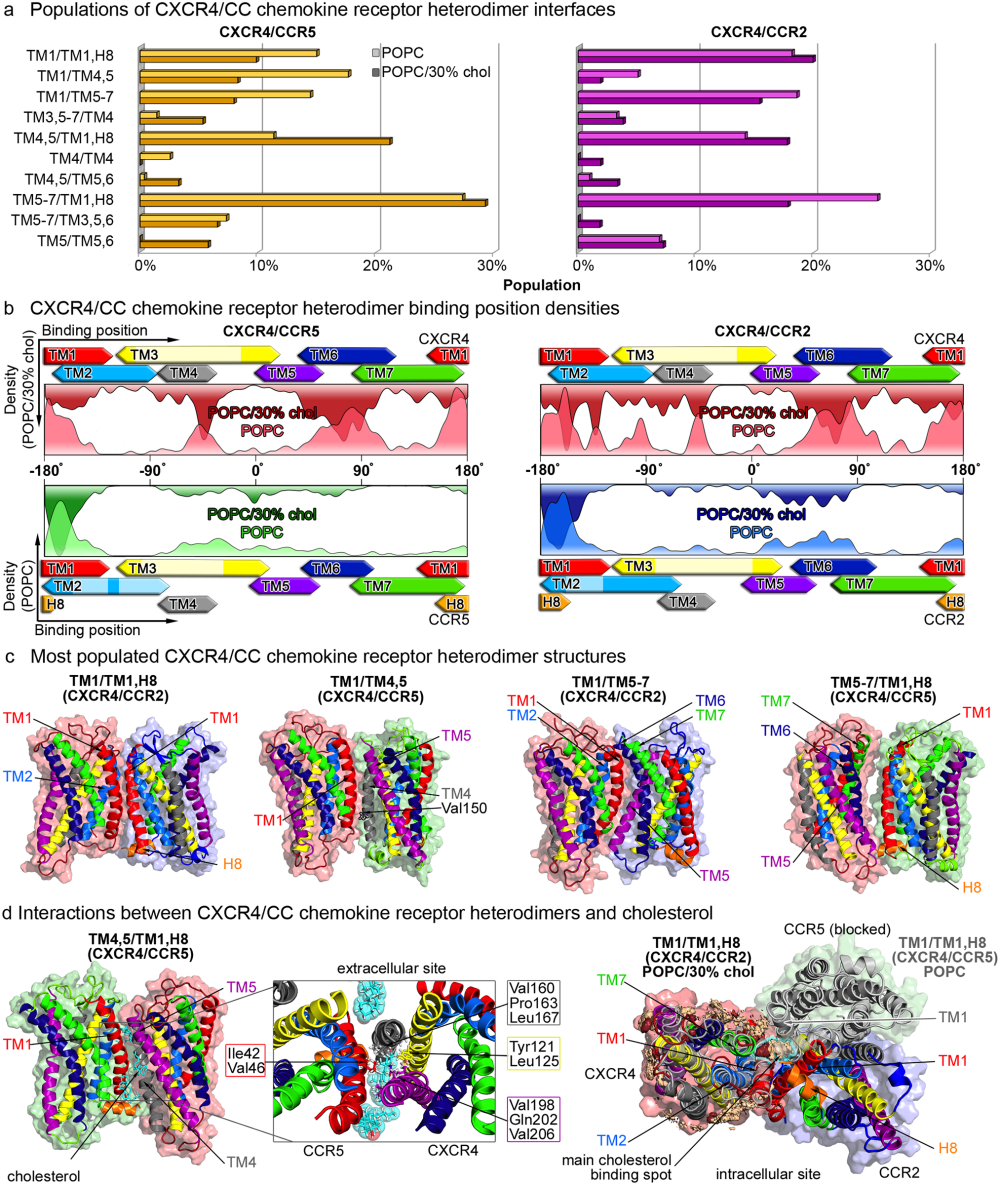
CXCR4/CC chemokine receptor heterodimer interfaces. **a** Populations of CXCR4/CC chemokine receptor heterodimers after 3 *μs* in pure POPC membranes (light colors), and after 8 *μs* in POPC membranes containing 30% cholesterol (dark colors). **b** Heterodimer binding position densities as introduced in Fig 3. Binding position densities on CXCR4 are shown in red, on CCR5 in green, and on CCR2 in blue. **c** Representative chemokine receptor heterodimer structures for the most populated interfaces. The protein surface is colored red for CXCR4, green for CCR5, and blue for CCR2. Transmembrane helices are colored consistent with Fig 2. **d** Interactions between CXCR4/CC chemokine receptor heterodimers and cholesterol. Cholesterol molecules are shown as sticks and colored in cyan and the density distribution of cholesterol around the protein is colored in light orange. Residues interacting at the TM4,5/TM1,H8 CXCR4/CCR5 interface are enframed according to the transmembrane helix color they are located on. The density distribution of cholesterol at the main binding spot on CXCR4 between TM1 and TM7, impeding the formation of CXCR4/CCR5 heterodimers involving TM1 of CXCR4, is highlighted in cyan.

Fig 5a shows the populations of the dominant CXCR4/CC chemokine receptor heterodimers. As depicted, the TM1/TM1,H8 heterodimer interface was the only highly populated symmetric heterodimer interface, whereas most of the heterodimer complexes were formed by asymmetric interfaces involving mostly TM1, TM4, and TM5-7. Other symmetric interfaces (TM4/TM4 or TM5/TM5) showed rather small populations in both pure POPC and mixed POPC/30% cholesterol lipid bilayers (see S4b Fig for representative structures for less populated CXCR4/CC chemokine receptor heterodimer configurations). Similar to the homodimerization of CC chemokine receptors, TM1 served as a significant heterodimerization hotspot (see Fig 5b), in agreement with experimental studies [32, 65, 68].

Despite the high sequence identity between the transmembrane segments of CCR5 and CCR2, ranging from 72% to 92% for individual helices (see S1 Fig), several differences in their heterodimerization patterns with CXCR4 could be identified. CXCR4 and CCR5 frequently formed TM1/TM4,5 interfaces (especially in pure POPC membranes), however, this heterodimer conformation was only sparsely populated in case of CXCR4/CCR2 complexes. As shown in Fig 5c, the experimentally pinpointed Val150 of CCR5 [32] is involved at this interface (corresponding to Ile163 in case of CCR2).

Furthermore, the heterodimerization of CXCR4 and CCR5 revealed a higher cholesterol sensitivity as compared to the association of CXCR4 and CCR2. The response of CXCR4/CCR5 heterodimerization to the addition of cholesterol was comparable to its influence on the homodimerization of CXCR4 [47]. I.e., the involvement of TM1 of CXCR4 was impaired due to cholesterol binding to the main binding spot between TM1 and TM7 of CXCR4 (see Fig 2a). In contrast, the number of dimers involving TM4 of CXCR4 at the interface significantly increased in cholesterol-rich membranes (see Fig 5b). As it can be seen, cholesterol molecules intercalated between the chemokine receptors to stabilize this interface.

In case of CXCR4/CCR2 heterodimers, cholesterol did not show an equally strong influence on receptor association and the populations of dimer interfaces remained rather stable in both lipid environments. Both the TM1/TM1,H8 and TM1/TM5-7 binding interfaces between the heterodimers are significantly shifted (see S5b1 and S5b2 Fig), displaying for TM1/TM1,H8 a rotation of ≈ 20° of CCR2 as compared to the corresponding CXCR4/CCR5 configuration (Fig 5d). Consequently, apart from TM1 also TM2 of CXCR4 contributes to heterodimerization with CCR2 (see Fig 5b and 5c), thereby restricting the influence of cholesterol-binding to TM1 on dimerization.

Supporting data was reported from BRET experiments: TM2 peptides derived from the sequence of TM2 of CXCR4 could be shown to reduce conformational changes in preexisting CXCR4/CCR2 heterodimers promoted by SDF-1 (agonist of CXCR4), thus hinting towards an involvement of TM2 in CXCR4/CCR2 dimers [35]. Additionally, other CXCR4 derived TM peptides—namely TM4, TM6, and TM7—strongly decreased the SDF-1-induced conformational changes in preexisting CXCR4/CCR2 heterodimers in HEK293T cells [35]. Furthermore, all four peptides modestly reduced the configurational response of CXCR4/CCR2 dimers upon treatment with MCP-1 (agonist for CCR2) [35]. The binding position densities on CXCR4 in CXCR4/CCR2 heterodimers (see Fig 5b) strongly correlate with the helices indicated by the experiments.

Our results elucidate how the heterodimerization of chemokine receptors can be regulated by membrane cholesterol enabling distinct dimerization patterns for sequentially highly related proteins. These findings suggest that heterodimerization and the interplay with the membrane environment play an important role in fine-tuning chemokine receptor signaling pathways.

#### Cholesterol-induced confinement of CCR5/CCR2 heterodimer interfaces

**Fig 6:**
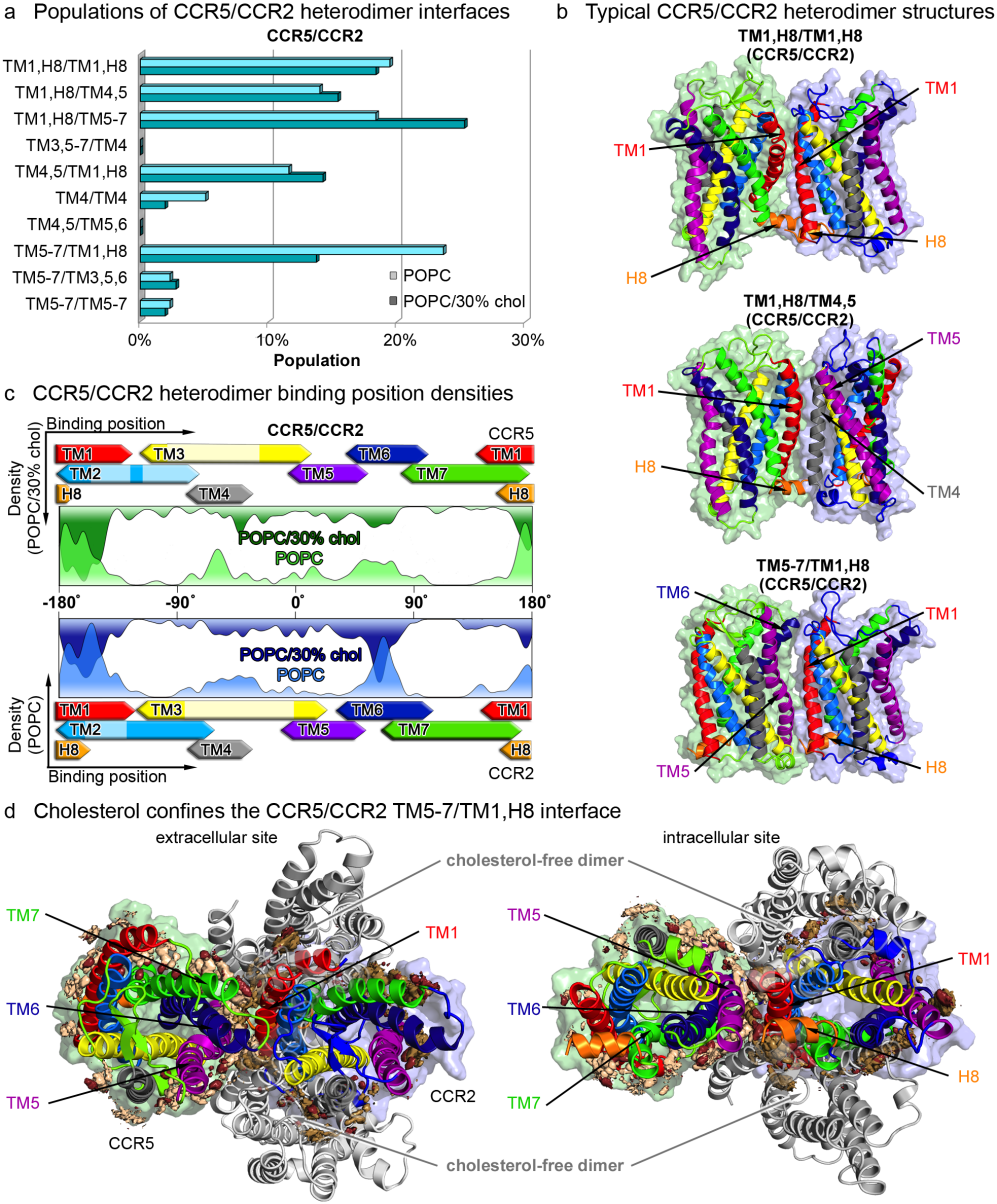
CCR5/CCR2 heterodimer interfaces. **a** Populations of CCR5/CCR2 chemokine receptor heterodimers after 3 *μs* in pure POPC membranes (light colors), and after 8 *μs* in POPC membranes containing 30% cholesterol (dark colors). **b** Representative chemokine receptor heterodimer structures for the most populated interfaces. The protein surface of CCR5 is colored in green and in blue for CCR2. Transmembrane segments are colored according to Fig 2. **c** Heterodimer binding position densities as introduced in Fig 3. Binding positions on CCR5 are colored in green and on CCR2 in blue. **d** The spatial distribution of cholesterol around the protein is colored in light orange. Cholesterol-free dimers (taken from the simulations in pure POPC) are shown in light grey.

Besides heterodimerization of CXCR4 with CCR5 or CCR2, the CC chemokine receptors have also been reported to associate with one another [34, 36, 42, 67, 68]. As shown in Fig 6a and 6b, especially TM1, TM4 and TM5 of both CC chemokine receptors serve as dimerization hotspots, supporting previous experimental findings [32, 65].

In general, the observed CCR5/CCR2 heterodimerization interfaces resemble the homodimer configurations of both CC chemokine receptors (see Fig 4 and S5b3 Fig, less populated CCR5/CCR2 heterodimer structures are shown in S4c Fig). Interestingly, TM5,6 and 7 of CCR2 revealed a stronger involvement in heterodimer interfaces with CCR5 as compared to CCR2 homodimerization or CCR2 heterodimerization with CXCR4 (see Fig 6c). Overall, similar to the above case of CC chemokine receptor homodimers, cholesterol did overall not show a strong impact on CCR5/CCR2 heterodimerization.

However, cholesterol confined the observed configurational flexibility of heterodimers in the TM5-7/TM1,H8 interface: In pure POPC bilayers, the CCR2 binding within this configuration covers a range of ≈ 50° (see Fig 6d and S5b3 Fig). In turn, binding of cholesterol decreased the population of TM5-7/TM1,H8 dimers and locked the dimer configuration in a specific relative orientation. This finding elucidates how cholesterol may act as a molecular *glue* to confine transmembrane protein association [21, 22, 47, 93].

## Discussion

During the last decade, an increasing number of studies revealed the role of homo- and heterodimerization of chemokine receptors in modulating protein function by affecting their chemokine sensitivity or altering their G protein coupling mechanisms [43, 48]. Besides protein function, the ability of the HI-Virus to infect T cells abusing either CXCR4 or CCR5 as a coreceptor was shown to depend on homo- and heterodimerization of the chemokine receptors [28, 34, 45, 68]. Consequently, targeting the association of chemokine receptors moved into the focus of modern drug discovery. The development of either bivalent ligands [71] or TM peptides [27, 32, 35, 53, 65] to regulate chemokine receptor dimerization and function is largely based on structural information of receptor dimer structures. Since structural detail of GPCR dimer complexes is rather limited due to experimental restrictions, we employed extensive molecular dynamics simulations to investigate the dimerization process and gain first molecular insight into homo- and heterodimer structures of chemokine receptors in lipid bilayers. POPC lipid bilayers serve as a robust membrane mimetic to study the interactions between membrane components in a controlled environment [20]. Based on the observation that cholesterol plays an important role in regulating chemokine receptor function and dimerization [53, 55–63], the effect of cholesterol on the protein association was additionally studied.

Using ensembles of simulations, similar homodimerization patterns were observed for the closely related CC chemokine receptors CCR5 and CCR2, distinct from the homodimerization interfaces of CXCR4 [47]. Especially TM1 of CC chemokine receptors served as a homodimerization hotspot as suggested before by experiments [32, 65]. TM1,H8/TM1,H8 interactions formed the only highly populated symmetric CC chemokine receptor homodimer interface, while other dimers were formed mostly via asymmetric TM1,H8/TM4,5 or TM1,H8/TM5-7 contacts. Cholesterol molecules bound to homologous regions on the CC chemokine receptors that differed from the main cholesterol binding sites on CXCR4. Residues indicated by experiments to play crucial roles in regulating CC chemokine receptor dimerization and function, namely Ile52 and Val150 on CCR5 as well as Val64 and Ile163 on CCR2 [32], were both located at the spontaneously formed dimer interfaces and within cholesterol binding sites on the receptor surfaces. These findings substantiate earlier experimental studies [32, 53, 58, 65], that residues which are not directly involved at the ligand- or G protein binding sites but exposed at the receptor surface can modulate GPCR function either via dimerization or interactions with the membrane environment.

Interestingly, the presence of cholesterol showed distinct effects on the homodimerization patterns of the three chemokine receptors: CXCR4 homodimer configurations involving TM1 were impaired in cholesterol-rich membranes, while TM4 was more frequently involved at dimer interfaces [47]. Even though cholesterol showed a smaller influence on the homodimerization patterns of both CC chemokine receptors as compared to CXCR4, the dimerization response differed between the closely related receptors. Thus, cholesterol can increase the dynamic range and versatility of associations of closely related transmembrane receptors.

Besides homodimerization of chemokine receptors, heterodimerization was studied in cholesterol-free and -rich membranes. The CXCR4/CC chemokine receptor heterodimerization revealed mostly asymmetric dimer configurations involving mainly TM1, TM4 and TM5-7 helices of each protomer at the interface but also symmetric TM1/TM1 dimer configurations. Interestingly, despite the high sequence identity between CCR5 and CCR2, differences were observed for their heterodimerization with CXCR4. Involvement of TM2 of CXCR4 in CXCR4/CCR2 heterodimer interfaces was enhanced as compared to CXCR4/CCR5 complexes. For the latter, CCR5 predominantly bound to areas involving TM1 and TM7 of CXCR4. Experiments conducted by Percherancier *et al.* [35] using TM peptides derived from CXCR4 showed that TM2 and TM4 peptides strongly reduced SDF-1-promoted conformational changes in preexisting CXCR4/CCR2 heterodimers, indicating that these helices are indeed involved at heterodimeric interfaces.

The subtle differences between CXCR4/CC chemokine receptor heterodimer configurations regarding the involvement of TM2 of CXCR4 at dimer interfaces with CCR2 turned out to have a significant impact on the cholesterol-sensitivity of the receptor association: binding of cholesterol to the main cholesterol binding site on CXCR4 at the intracellular halves of TM1 and TM7 hampered the formation of CXCR4/CCR5 heterodimer interfaces formed around TM1 of CXCR4, while it did not impede corresponding CXCR4/CCR2 conformations. Interestingly, the heterodimerization response of CXCR4/CCR5 complexes to cholesterol showed the same mechanism as described for CXCR4 homodimers [47], i.e. interfaces involving TM1 were reduced while the ratio of configurations including TM4 of CXCR4 increased.

As discussed in the Introduction, CXCR4 and CCR5 have both been observed to require cholesterol for proper function [55–59]. Furthermore, the HIV-entry via virus-binding to CD4 and either of the coreceptors CXCR4 or CCR5 was reduced upon depletion of cholesterol [57, 60, 61] and upon heterooligomerization between CXCR4, CCR5 and CD4 in T cells [45]. Our data suggest a cholesterol-sensitive heterodimerization of CXCR4 and CCR5. It appears intriguing to hypothesize that the CXCR4/CCR5 heterodimer interfaces predominantly formed in cholesterol-containing membranes, i.e. TM4,5/TM1,H8 and TM5-7/TM1,H8 dimers, interact with CD4 and display resistance to HIV-entry.

In conclusion, associations between the chemokine receptors CXCR4, CCR5 and CCR2 and their interaction with cholesterol, were reported to play essential roles in diversifying receptor function. Here, we provide a structural basis for the versatile interplay between the receptor homo- and heterodimerization and its modulation by cholesterol, aimed to contribute to the deciphering of the complex regulation network of chemokine related signaling pathways and diseases.

## Materials and methods

### Structure preparation

The protein structure of CXCR4, based on the crystal structure 3OE0 [77] was taken from our previous study [47]. The preparation of the CC chemokine receptors CCR5 and CCR2 followed a similar protocol. The crystal dimer structure of the chimera protein containing two copies of CCR5, rubredoxin, and the antagonist drug maraviroc [79] was downloaded from the Protein Data Bank (PDB entry 4MBS). One monomer, maraviroc, rubredoxin, and other non-protein atoms were removed. The thermostabilizing mutations in the CCR5 crystal structure—Tyr58Cys, Asn163Gly, Asp233Ala and Glu303Lys—were mutated back to the wildtype sequence and missing residues of the third intracellular loop, namely Cys224, Arg225, and Asn226, were added to the protein structure (using MODELLER [110]). The final structure contained residues ranging from Pro19 to Phe312, the terminal parts of the protein were not resolved in the crystal structure. The isoform B of CCR2 was crystallized in a chimeric complex with T4-lysozyme, an orthosteric and an allosteric antagonist (BMS-681 and CCR2-RA-[R], respectively) [80]. Thereby, the wildtype residues ranging from Leu226 to Arg240 were removed and replaced with a sequence derived from the M2 muscarinic acetylcholine receptor in order to fuse with the T4-lysozyme at the position of the third intracellular loop [80]. Here, during the structure preparation, both antagonists, the T4-lysozyme and other non-protein atoms were removed from the crystal structure obtained from the Protein Data Bank (PDB entry 5T1A). Next, the third intracellular loop was modelled according to the native sequence, i.e. Leu226—KTLLRCRNEKKRHR—Arg240. Since both termini were not resolved in the crystal structure, the resulting CCR2 structure contained residues from Val37 to Phe320. Finally, both CC chemokine receptor structures were minimized using the CHARMM36 force field [111].

**Fig 7:**
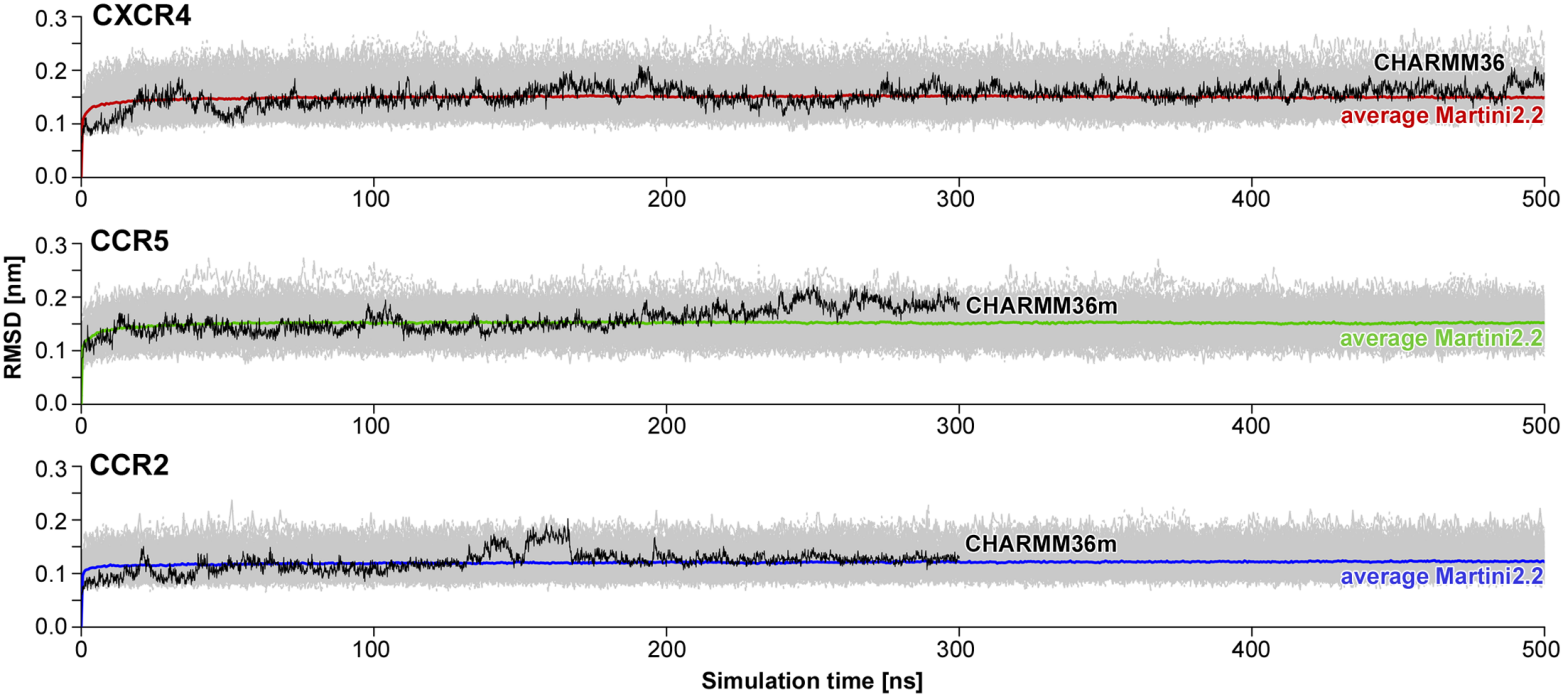
Comparison of protein root mean square deviations (RMSD) between coarse-grained and atomistic simulations. RMSDs of transmembrane helix backbone beads determined from 100 coarse-grained (CG) homodimerization simulations (initial 500 ns) in POPC membranes (for systems where no interaction energies between both monomers were observed until 1 *μs*, shown in light grey). The average RMSDs of CG simulations are colored in red for CXCR4, green for CCR5, and blue for CCR2. The RMSD curves for atomistic simulations (CHARMM36 [111] for CXCR4 and CHARMM36m [116] for CC chemokine receptors) of corresponding chemokine receptor monomers in POPC membranes, shown in black, were calculated by converting the atomistic simulation frame-by-frame (time step of 100 ps) to the Martini2.2 force field in order to enable a direct comparison to the CG simulations. The data for CXCR4 was taken from our previous study [47].

Subsequently, the protein structures were converted to the Martini2.2 coarse-grained force field [112] using *martinize* [112]. In order to enforce the secondary and tertiary structure, an elastic *RubberBands* force network was applied. *RubberBands* as established in Wassernaar *et al.* [82] introduce additional weak bonds (with force *F*) between all backbone bead pairs (*i*, *j*) within a distance (*d_ij_*) of 0.9 nm following the kernel like function [113]: $F_{ij}=f\cdot e^{-ad_{ij}^{2p}}$. Here, *f* was set to 500 kJ/mol/nm^2^, *a* was set to 3, and *p* to 6. Thereby, beads that are by default connected via bonds and angles within the Martini force field, i.e. *i* − *i* + 1 and *i* − *i* + 2 backbone bead pairs were excluded from the *RubberBand* network. *RubberBands* is, as well as ElNeDyn [114], implemented in *martinize* [112] and was chosen here, since it allows to run simulations using the standard Martini protein force fields [112, 115]. The application of *RubberBands* on the coarse-grained structures did not enhance the protein rigidity artificially as it can be seen from a comparison of the RMSD values between CG and atomistic simulations in Fig 7.

### Simulation setup

#### Coarse-grained simulations

The homo- and heterodimerization simulations were setup using the DAFT protocol [82]. In every simulation, two CG protein structures were placed at the same defined initial distance (approx. 3.5 nm minimum distance) into a rhombic box, whereby, both proteins were randomly rotated around the z-axis (membrane normal) resulting in different relative orientation for every starting state of the simulations. Subsequently, CG lipid bilayers [117] consisting either exclusively of POPC lipids or containing POPC and cholesterol at a 7:3 ratio as well as CG water [118] were added using *insane* [119]. The final systems contained two receptors, approx. 6,000–6,500 water beads and roughly 360 POPC or 320 POPC and 140 cholesterol molecules in case of pure POPC or mixed POPC/cholesterol membranes, respectively.

In addition, systems containing only one receptor embedded in POPC membranes with 10% cholesterol content were set up using DAFT in order to study specific interactions between the protein and cholesterol. These systems contained one receptor, roughly 220 POPC lipids, 25 cholesterol molecules, and 4,500 water beads.

Following the *martinate* protocol [120], each system was minimized for 1,500 steps with the steepest-descent algorithm before undergoing a 100 ps *NPT* equilibration MD simulation with a 20 fs time step at 310 K and 1 bar. The final production runs were performed in *NPT* ensembles with a MD integration time step of 20 fs where the system’s center of mass movement was removed every 10 steps. Using the v-rescale thermostat [121] with a coupling time constant of 1 ps, the temperature was kept at 310 K and the pressure was controlled at 1 bar applying the Berendsen barostat [122] in a semi-isotropical manner (xy dimensions were scaled independently from the z dimension, i.e. the membrane normal) with a coupling time constant of 3 ps. As suggested for the standard Martini force field, the relative dielectric constant was set to 15 [118]. Electrostatic Coulomb interactions were shifted to zero between 0 and 1.2 nm and the 12-6 Lennard-Jones potential describing the van der Waals forces was shifted to zero between 0.9 and 1.2 nm.

All coarse-grained simulations were performed in GROMACS 4.6 [123] in order to be consistent with the previous study regarding the homodimerization of CXCR4 [47].

#### Atomistic simulations

Atomistic simulations of CC chemokine receptor monomers in POPC membranes were setup according to the following protocol: First, a coarse-grained Martini2.2 protein structure was embedded in a hexagonal POPC membrane using *insane* [119]. The CG systems contained roughly 320 POPC lipids and 5,300 water beads. The systems were minimized for 500 steps using the steepest descent algorithm and subsequently equilibrated in the *NPT* ensemble by performing short MD simulations with position restraints on the protein structure. These simulations were performed for 100 ns using the same simulation parameters as the final production runs of the dimerization simulations.

In the next step, the systems were converted back to atomistic detail using *backward* [124] in order to prepare the system for simulations with the CHARMM36m force field [116, 125]. Ions were added to reach a salt concentration of 0.15 nM and to set the system net charge to zero. Prepared atomistic systems contained roughly 320 lipids, 21,000 water molecules and 130 ions. The corresponding prepared protein crystal structure were fitted onto the backmapped structure and energetically minimized for 1,000 steps with the steepest descent algorithm. Subsequently, the systems were simulated in the *NPT* ensemble with position restraints on the protein backbone for 50 ns with an MD integration time step of 2 fs. The temperature of 310 K was kept by applying the v-rescale thermostat [121] with a coupling time constant of 0.5 ps. The pressure of 1 bar was controlled using the Parrinello-Rahman pressure coupling [126] in a semi-isotropical manner with a time constant of 5 ps. The 12-6 Lennard-Jones potential was smoothly switched to zero between 0.8 and 1.2 nm. Electrostatic interactions were computed using the particle-mesh Ewald summation [127] for long-range interactions between particles separated by more than 1.2 nm. Finally, the position restraints were removed and the systems were simulated for 300 ns using the same simulation parameters as in the position restraint simulations.

All atomistic simulations were performed in GROMACS 5 [128].

### Analysis

#### Dimerization criterium and coarse-grained lower-bound binding free energy estimates

**Fig 8:**
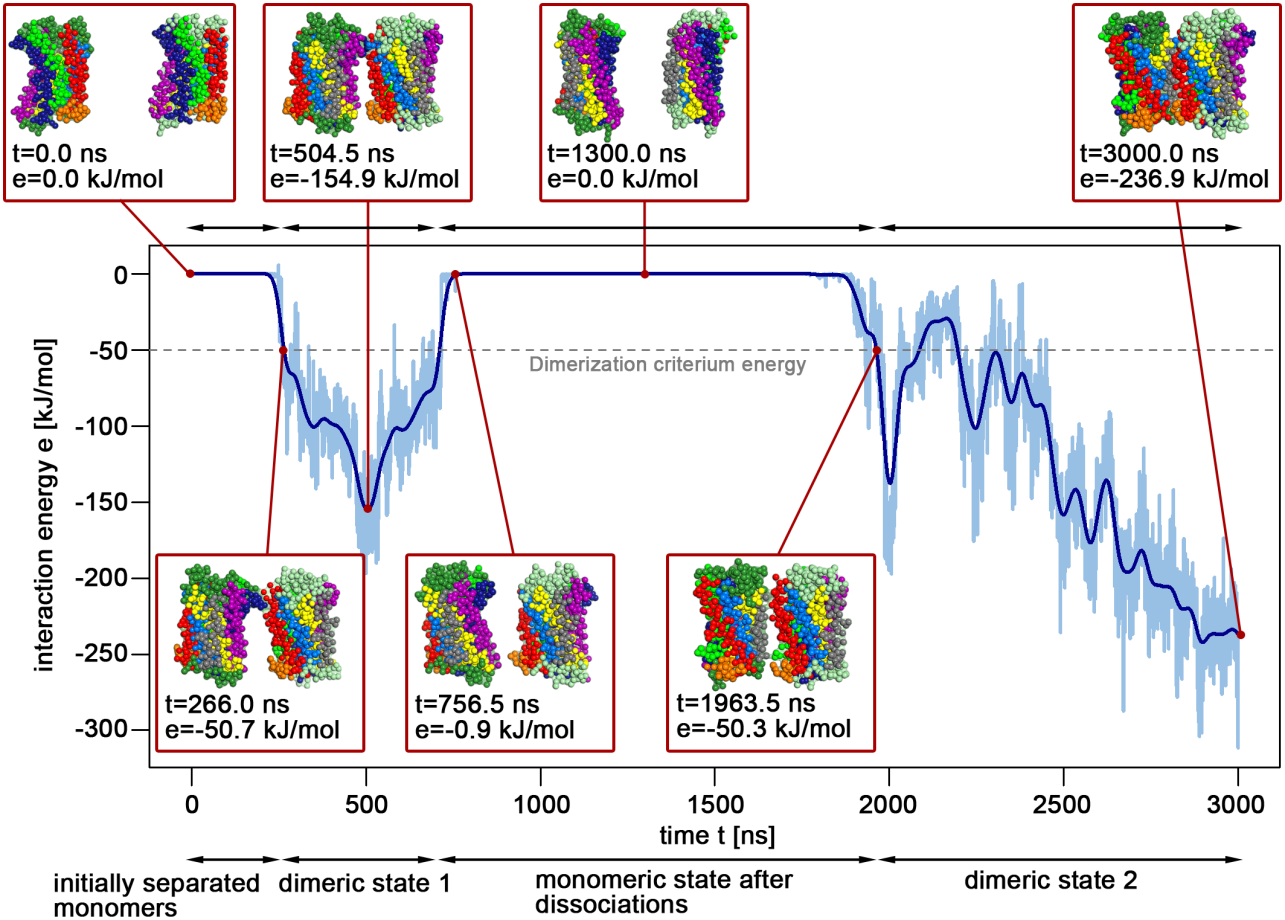
Interaction energy profile with representative snapshots. The energy profile shows the interaction energy (estimated as the sum of Lennard-Jones and Coulomb interaction energies) between the transmembrane helices of two CCR5 proteins in a pure POPC membrane during a 3 *μs* simulation.

As established in our previous study regarding the homodimerization of CXCR4 [47], two interacting receptors were considered as a dimer if their interaction energy (estimated as the sum of Lennard-Jones and Coulomb interaction energies) between their TM helices was lower than -50 kJ/mol. This simple energy cut-off was chosen to distinguish protein monomers from dimers, since receptors interacting at -50 kJ/mol revealed direct protein-protein contacts (see Fig 8). Dissociation events (see Fig 1b and S2d Fig) were observed if the interaction energy between proteins changed from -50 kJ/mol to more than -1 kJ/mol. Coarse-grained lower-bound binding free energy estimates, $\Delta G_{lower-bound}^{cg}$, were computed following the approach proposed by de Jong *et al.* [129]. Accordingly, the total ensemble simulation time was divided in times spent in dimeric states or monomeric states, dissociated from dimeric states [130] (see S2 Fig). In this study, we computed dissociation propensities and estimated lower-bound free energy estimates only for the three most populated homodimer interfaces or five most populated heterodimer interfaces, in order to exclude possible unreasonable dimer formations. In order to assess binding affinity and dissociation for increasingly compact dimers, dissociation propensities and $\Delta G_{lower-bound}^{cg}$ were additionally calculated for the dimerization criteria -100 kJ/mol, -150 kJ/mol and -200 kJ/mol (see S3 Fig).

#### Orientation analysis

**Fig 9:**
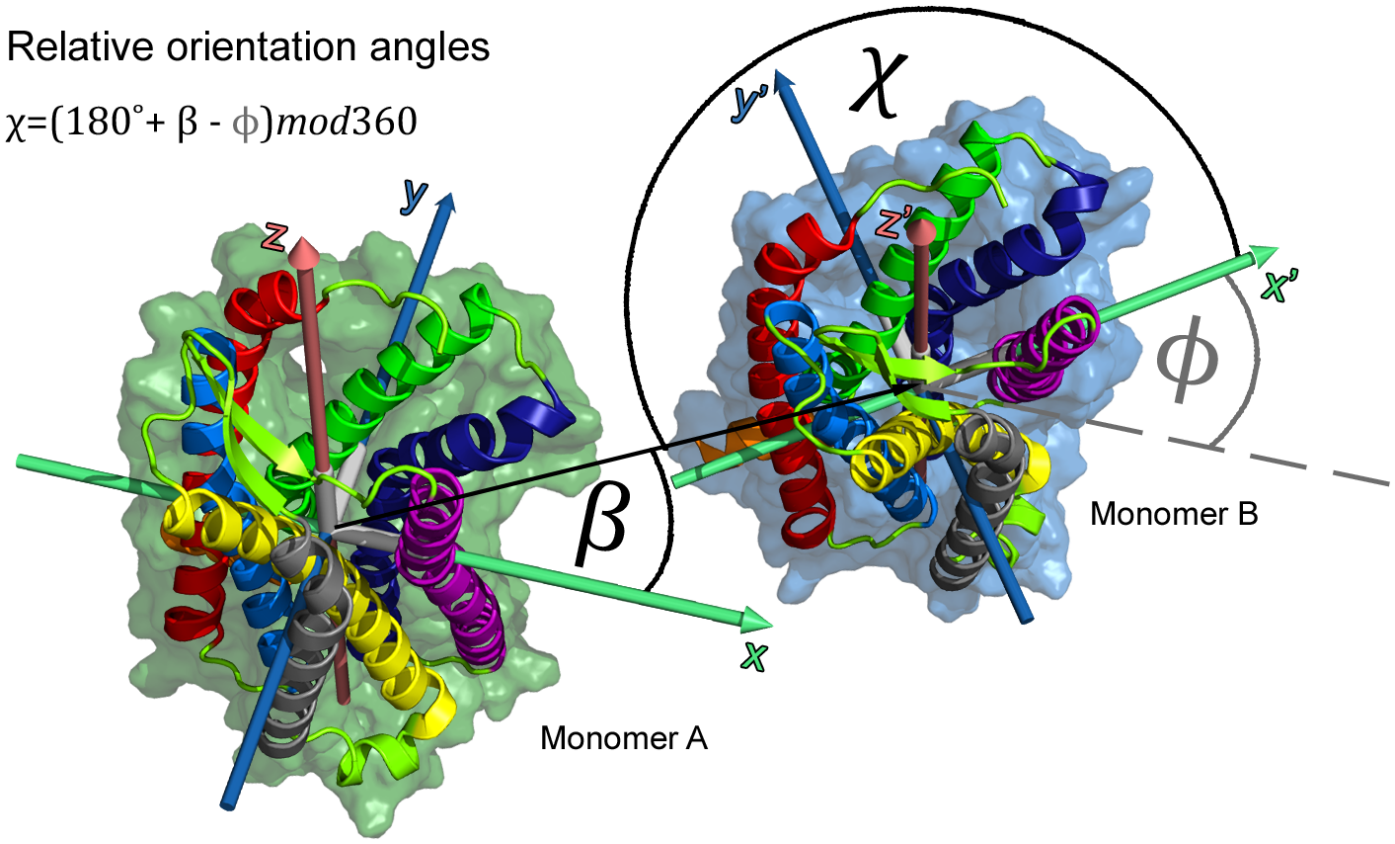
Relative orientation angles at dimer interfaces. Three relative angles are calculated to describe dimer configurations: *β* describes the position of binding of monomer B to the reference structure monomer A given as the rotation of monomer B around the z-axis of monomer A. The angle *ϕ* defines the rotation of monomer B around its’ own z-axis (z’) and *χ* = (180° + *β* − *ϕ*)*mod*360 describes the position of binding of monomer A to monomer B.

Dimer interfaces were determined by investigating the relative orientation between monomers in dimer configurations. As described in previous studies [47, 82], each protein structure in a simulation was assigned a center of mass and an internal coordinate system according to its’ principal components. Relative angles between proteins, as shown in Fig 9, were determined by superimposing their coordinate systems. The binding site of the interaction partner (monomer B) on the reference protein (monomer A) is described by the angle *β*, i.e. the rotation of the interaction partner around the z-axis of the reference structure. In turn, the binding site of the reference structure on the interaction partner is defined by the angle *χ* = (180° + *β* − *ϕ*)*mod*360, where *ϕ* describes the rotation of the interaction partner around its’ own z-axis.

Relative binding angles, *β* and *χ* were computed for spontaneously formed dimers for the last 50 ns of the simulations and kernel density height-fields of the resulting (*β*, *χ*)-coordinates, as shown in S5 Fig, were prepared. In the next step, the angle height-fields were investigated with the image processing watershed method [131] in oder to determine the spreading of height-field maxima and to assign every simulation frame to a specific maximum (see more details in [47]).

#### Binding position densities

The binding position densities shown in Figs 3, 5c and 6b were calculated from the relative orientation angles described in the previous section by integrating all contributions to a given orientational angle, i.e. *β* or *χ*. In case of homodimerization between chemokine receptors, the *β* and *χ* angles correspond to the same binding position on a receptor. Consequently, the binding position densities for homodimers were computed from all *β* and *χ* angles together. In case of heterodimers, *β* and *χ* describe binding positions on different receptors, e.g. as it can be seen in S5b1 Fig, *β* yields the binding position of CCR5 on CXCR4 while *χ* describes the binding site of CXCR4 on CCR5. The labels of the x-axes in Figs 3, 5c and 6b assign the binding position angles on a receptor to its’ transmembrane helices. The helix angles were computed as the orientations around the z-axis of helix backbone bead coordinates projected onto the xy-plane of the coordinate system constructed by the protein’s principal axes (see Fig 3b).

#### Identifying representative dimer structures

For the most populated dimer configurations, representative structures were selected in order to investigate corresponding dimer interfaces at atomistic detail. These structures fulfilled the following criteria: the final (*β*, *χ*)-coordinates were closest to the corresponding height-field maximum (see S5 Fig) while showing strong interaction energies between the monomers indicating a compact interface. Selected systems were converted back to atomistic resolution using *backward* [124] and further prepared with the CHARMM36m force field [116]. Ions were added to reach a salt concentration of 0.15 nM and to counteract the systems net charge. The prepared systems contained roughly 360 POPC lipids (pure POPC membranes) or 320 POPC and 140 cholesterol molecules (mixed POPC/30% cholesterol membranes), 26,000 water molecules and 150 ions. Subsequently, each system was minimized for 500 steps with the steepest descent algorithm and simulated for 10 ns with position restraints on the protein backbones using the same parameters as described for the atomistic simulations above.

#### Cholesterol binding sites

Binding of cholesterol to protein monomers was investigated by computing the occupancy of helix residues by cholesterol molecules. Cholesterol was considered as bound to a residue if at least one of the 8 cholesterol beads was located within 0.62 nm of the residue (as described in [47]). The spatial distributions of cholesterol around the protein were calculated for the five nearest cholesterol molecules. In addition, the spatial distributions of the polar headgroup beads (ROH) were computed from the five closest molecules. For every receptor, the data was taken from ten 1 *μs* coarse-grained simulations of a monomer embedded in a POPC membrane with 10% cholesterol content, discarding the initial 200 ns. Cholesterol binding to receptor dimers was analyzed using the spatial distributions of cholesterol and its headgroup around each receptor monomer (for five closest cholesterol molecules). Spatial distributions were calculated for the last 200 ns from all DAFT simulations of a specific dimer interface.

## Supporting information

S1 Fig Sequence alignments and percent identity matrices of CXCR4, CCR5, and CCR2. **a** Sequence alignment of protein segments resolved in the prepared crystal structures [77, 79, 80] (see Materials and methods). Transmembrane helices are enframed and colored according to the scheme presented in Fig 2. Sequences were aligned using the *Clustal Omega* algorithm [132] provided by the European Bioinformatics Institute web server [133, 134]. **b** Percent identities were calculated for the whole protein sequences and for the sequence presented in **a**. **c** Transmembrane helix sequence identities were calculated for the segments enframed in **a**.
(TIF)

S2 FigEnergies and kinetics of homo- and heterodimerization.**a** Number of dimers formed in the simulation ensemble. The data for the homodimerization of CXCR4 was taken from our previous study [47]. **b** Derivation of first order dimerization reaction rates *k* from the concentration of receptor monomers as a function of simulation time. The first order reaction results from the observation of systems in monomeric or dimeric conformation instead of the concentration of monomers and dimers in one system (which would result in a second order reaction). **c** Reaction rates *k* and parameters for estimating the lower bound binding free energies. *P_0_* /*P_1_* yields the ratio between the total simulation time in monomeric states (after dissociation) and in dimeric states. *V* denotes the volume of the protein-lipid bilayer. *K_D_* gives the estimated dissociation constant according to *K*_*D*_ = *P*_0_/*P*_1_ *c*^⊘^ *N*_*Av*_ *V* with a standard concentration of *c*^⊘^ = 1mol/l and the Avogadro constant *N_Av_* [129]. $\Delta G_{lower-bound}^{cg}$ estimates the lower bound for the binding free energy of the most populated dimer interfaces. **d** Absolute number of dissociation events from the most populated dimer interfaces.(TIF)

S3 FigDissociation propensities and coarse-grained lower-bound binding free energy estimates for increasing dimerization criteria interaction energies.Dissociation propensities were calculated as the ratio between the total number of dissociation events and the total number of dimerization events for the three or five most populated dimer interfaces of chemokine receptor homo- or heterodimers, respectively. Coarse-grained lower-bound binding free energy estimates, $\Delta G_{lower-bound}^{cg}$, were calcula ted as described in S2 Fig. For increasing dimerization threshold interaction energies (sum of Lennard-Jones and Coulomb interaction energies), less dissociations and thus increased binding free energy estimates are observed. Notably, cholesterol increased the dissociation propensities of every chemokine receptor combination using the lowest dimerization criterium (-50 kJ/mol), indicating a stronger effect of cholesterol on inital protein-protein contacts as compared to compact dimer interfaces (with higher interaction energy values).(TIF)

S4 Fig Representative structures of less populated chemokine receptor homo- and heterodimer interfaces. **a** CC chemokine receptor homodimers, **b** CXCR4/CC chemokine receptor heterodimers, and **c** CCR5/CCR2 heterodimers. The receptors are colored consistent with Fig 2.
(TIF)

S5 Fig Kernel density height-fields of two relative angles (*β*, *χ*) between monomers in dimeric structures. *β* denotes the binding position of monomer A on monomer B, whereas *χ* describes the angle under which monomer B binds to monomer A. Both angles were calculated for the last 50 ns for simulations in which dimers were formed. The most frequently observed (*β*, *χ*)-coordinates, i.e. dimer configurations, are labeled according to their corresponding dimer interfaces. **a** Densities of relative angles between monomers in chemokine receptor homodimers (**a**1: CXCR4 [47], **a**2: CCR5, **a**3: CCR2). **b** Densities of relative angles between monomers in chemokine receptor heterodimers (**b**1: CXCR4/CCR5, **b**2: CXCR4/CCR2, **b**3: CCR5/CCR2).
(TIF)

S6 Fig Structural comparison between the crystal dimer and the self-assembled TM1/TM4,5 dimer. **a** Top view (extracellular site) onto the crystal packing observed for the CCR5 receptor TM1,7/TM4,5 dimer shown in grey (PDB: 4MBS [79]). The dimer structure obtained from simulations is colored according to Fig 2. The structural alignment was performed only on monomer A. **b** Enlarged view of the structural fit. Intra- and extracellular loops are not shown for clarity.
(TIF)

S1 TableProtein self-diffusion coefficients.The lateral self-diffusion of proteins was calculated from the slope of the mean square displacement (MSD) averaged over the beads of each protein according to the Einstein relation:
$$\begin{array}{c} D_{s}=\lim_{t\to \infty }\frac{\left\langle \Delta r{\left(t\right)}^{2}\right\rangle }{4t} \end{array}$$
where Δ*r*(*t*) denotes the distance a bead moved in time *t*. The MSD was calculated for the inital 200 ns of the simulations. In case of dimerization simulations, the MSD was calculated for simulations that did not show interaction energies between the monomers until 250 ns. The center of mass motion of the protein-membrane system was substracted during the MSD calculations. The slope of MSD curves was fitted on the time window between 5-20 ns. Data for CXCR4 was taken from Pluhackova *et al.* [47].(PDF)
