# Supplementary figures and images for: Closely related, yet unique: Distinct homo- and heterodimerization patterns of G protein coupled chemokine receptors and their fine-tuning by cholesterol
Source: PLoS Comput Biol. 2018 Mar 12;14(3):e1006062. doi: 10.1371/journal.pcbi.1006062 (PMC5864085; doi:10.1371/journal.pcbi.1006062)

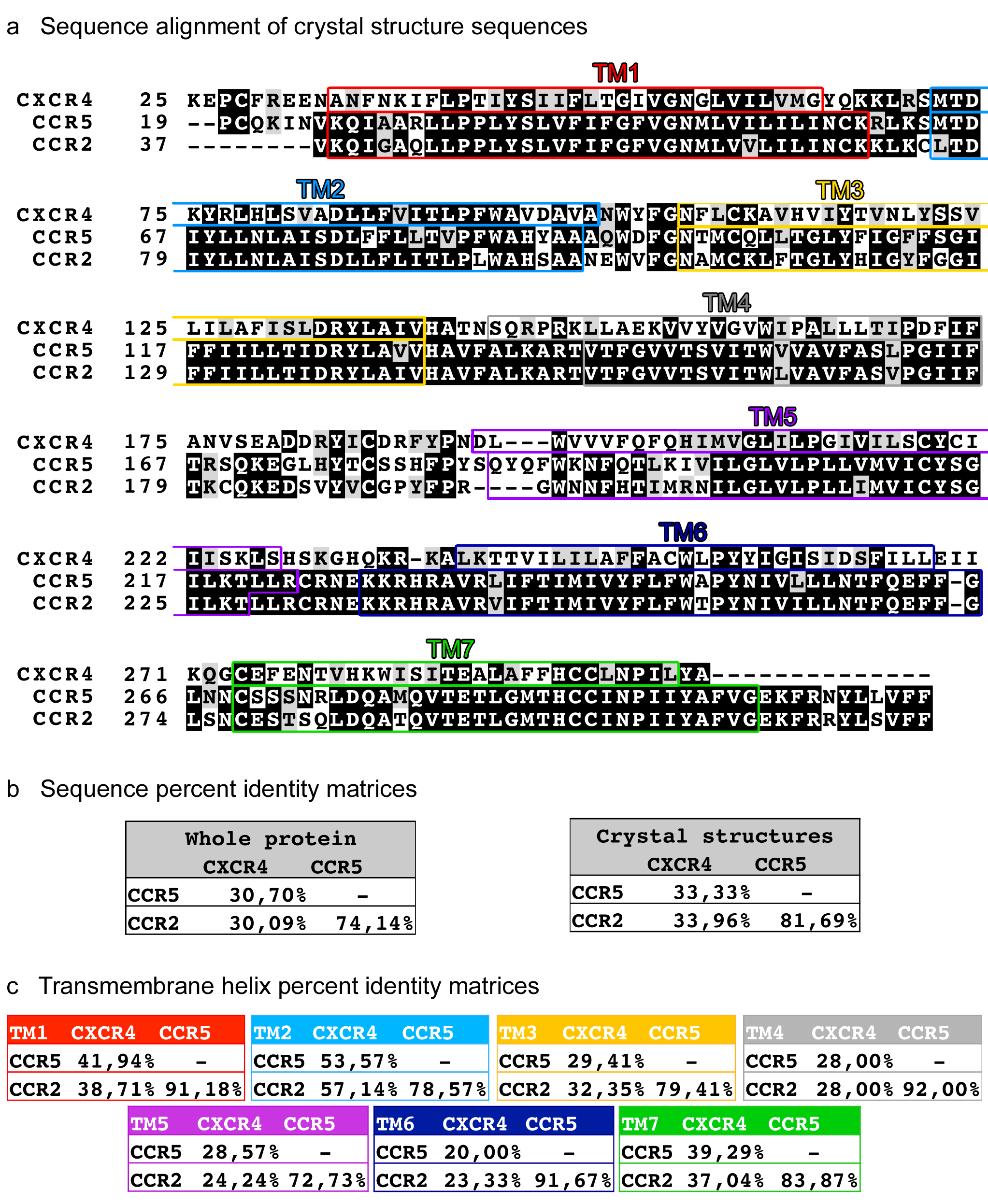

Supplement: S1 Fig — a Sequence alignment of protein segments resolved in the prepared crystal structures [77, 79, 80] (see Materials and methods). Transmembrane helices are enframed and colored according to the scheme presented in Fig 2. Sequences were aligned using the Clustal Omega algorithm [132] provided by the European Bioinformatics Institute web server [133, 134]. b Percent identities were calculated for the whole protein sequences and for the sequence presented in a. c Transmembrane helix sequence identities were calculated for the segments enframed in a. (TIF) [file pcbi.1006062.s001.tif]

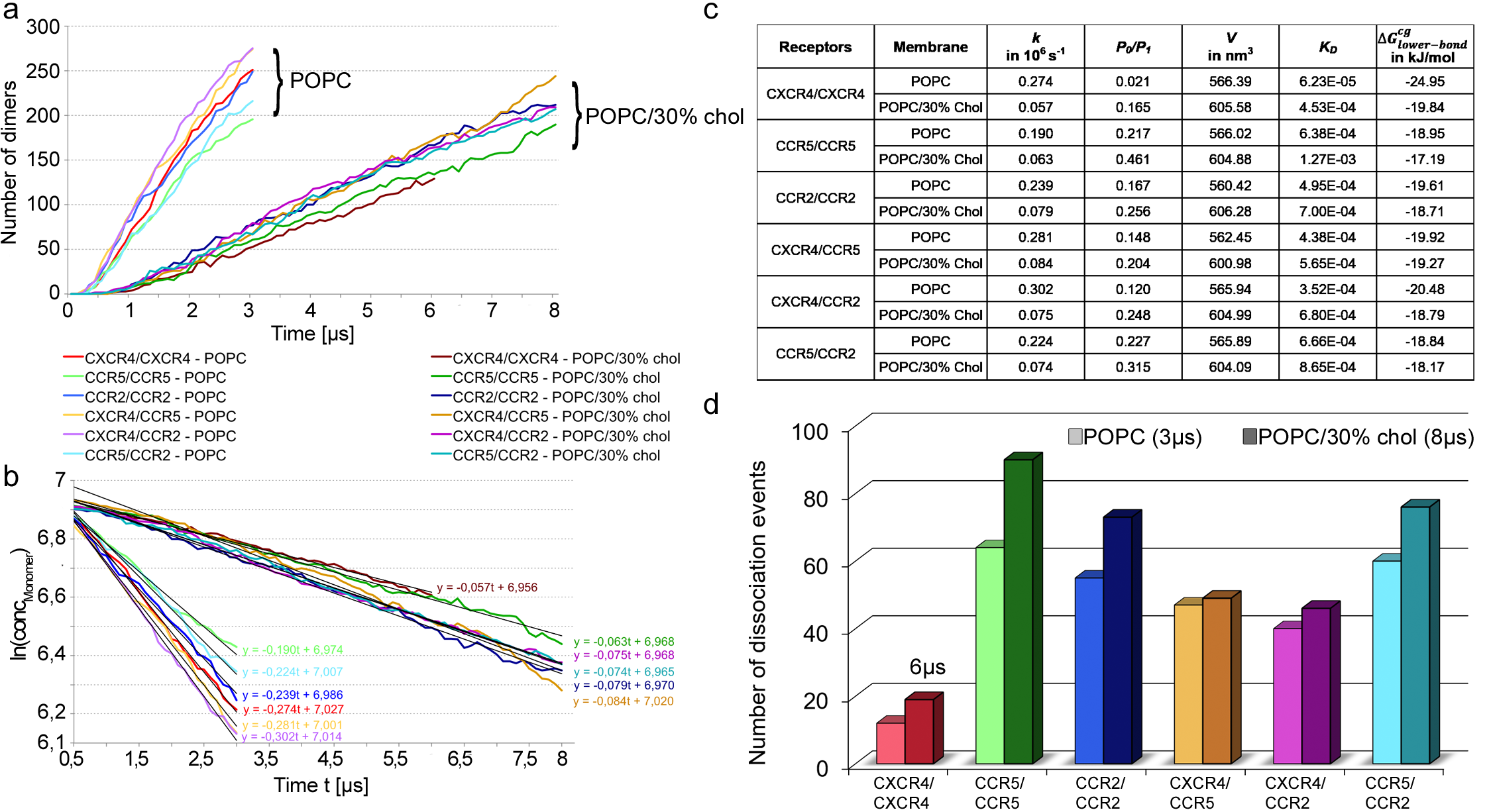

Supplement: S2 Fig — a Number of dimers formed in the simulation ensemble. The data for the homodimerization of CXCR4 was taken from our previous study [47]. b Derivation of first order dimerization reaction rates k from the concentration of receptor monomers as a function of simulation time. The first order reaction results from the observation of systems in monomeric or dimeric conformation instead of the concentration of monomers and dimers in one system (which would result in a second order reaction). c Reaction rates k and parameters for estimating the lower bound binding free energies. P0 /P1 yields the ratio between the total simulation time in monomeric states (after dissociation) and in dimeric states. V denotes the volume of the protein-lipid bilayer. KD gives the estimated dissociation constant according to KD = P0/P1 c⊘ NAv V with a standard concentration of c⊘ = 1mol/l and the Avogadro constant NAv [129]. ΔGlower-boundcg estimates the lower bound for the binding free energy of the most populated dimer interfaces. d Absolute number of dissociation events from the most populated dimer interfaces. (TIF) [file pcbi.1006062.s002.tif]

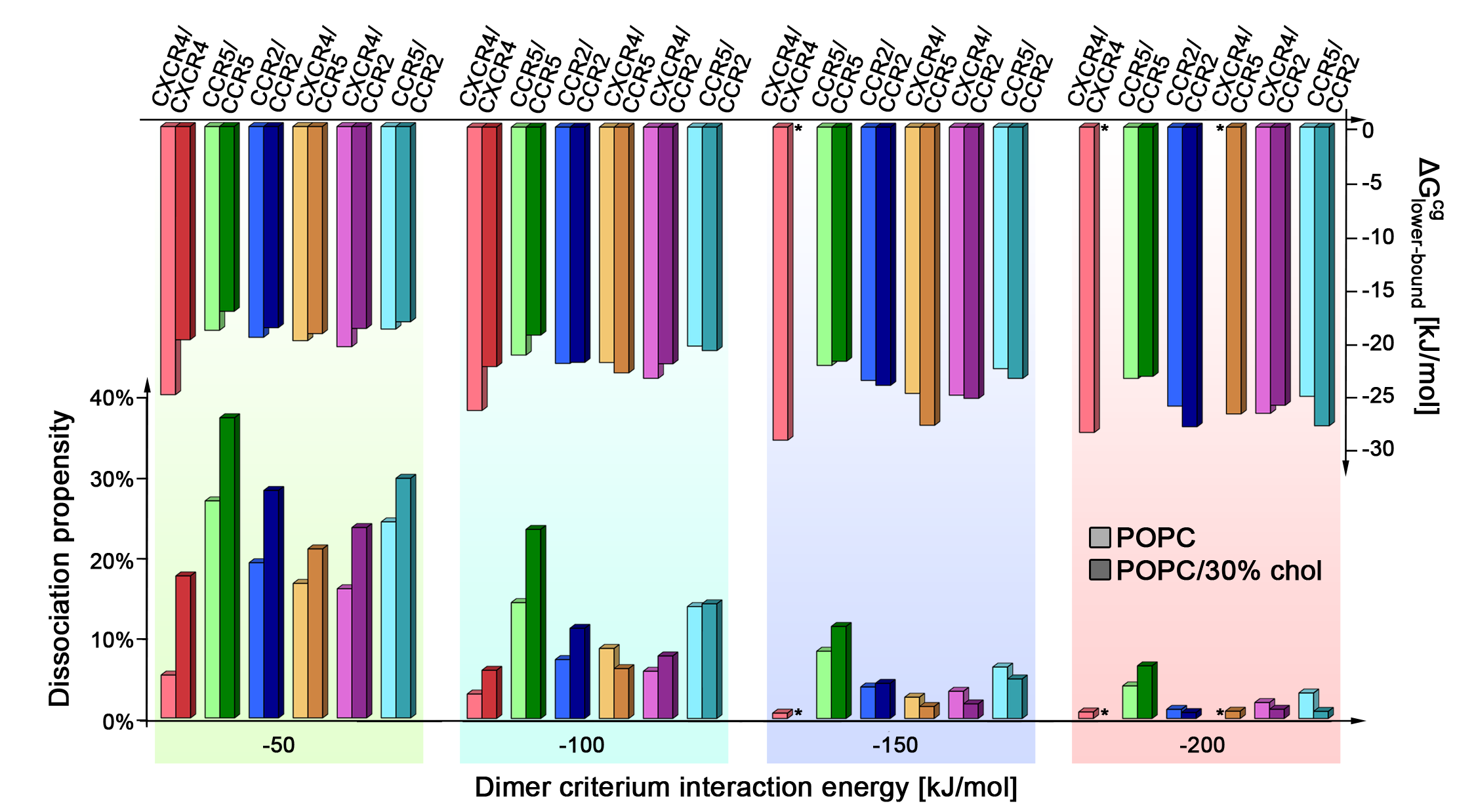

Supplement: S3 Fig — Dissociation propensities were calculated as the ratio between the total number of dissociation events and the total number of dimerization events for the three or five most populated dimer interfaces of chemokine receptor homo- or heterodimers, respectively. Coarse-grained lower-bound binding free energy estimates, ΔGlower-boundcg, were calcula ted as described in S2 Fig. For increasing dimerization threshold interaction energies (sum of Lennard-Jones and Coulomb interaction energies), less dissociations and thus increased binding free energy estimates are observed. Notably, cholesterol increased the dissociation propensities of every chemokine receptor combination using the lowest dimerization criterium (-50 kJ/mol), indicating a stronger effect of cholesterol on inital protein-protein contacts as compared to compact dimer interfaces (with higher interaction energy values). (TIF) [file pcbi.1006062.s003.tif]

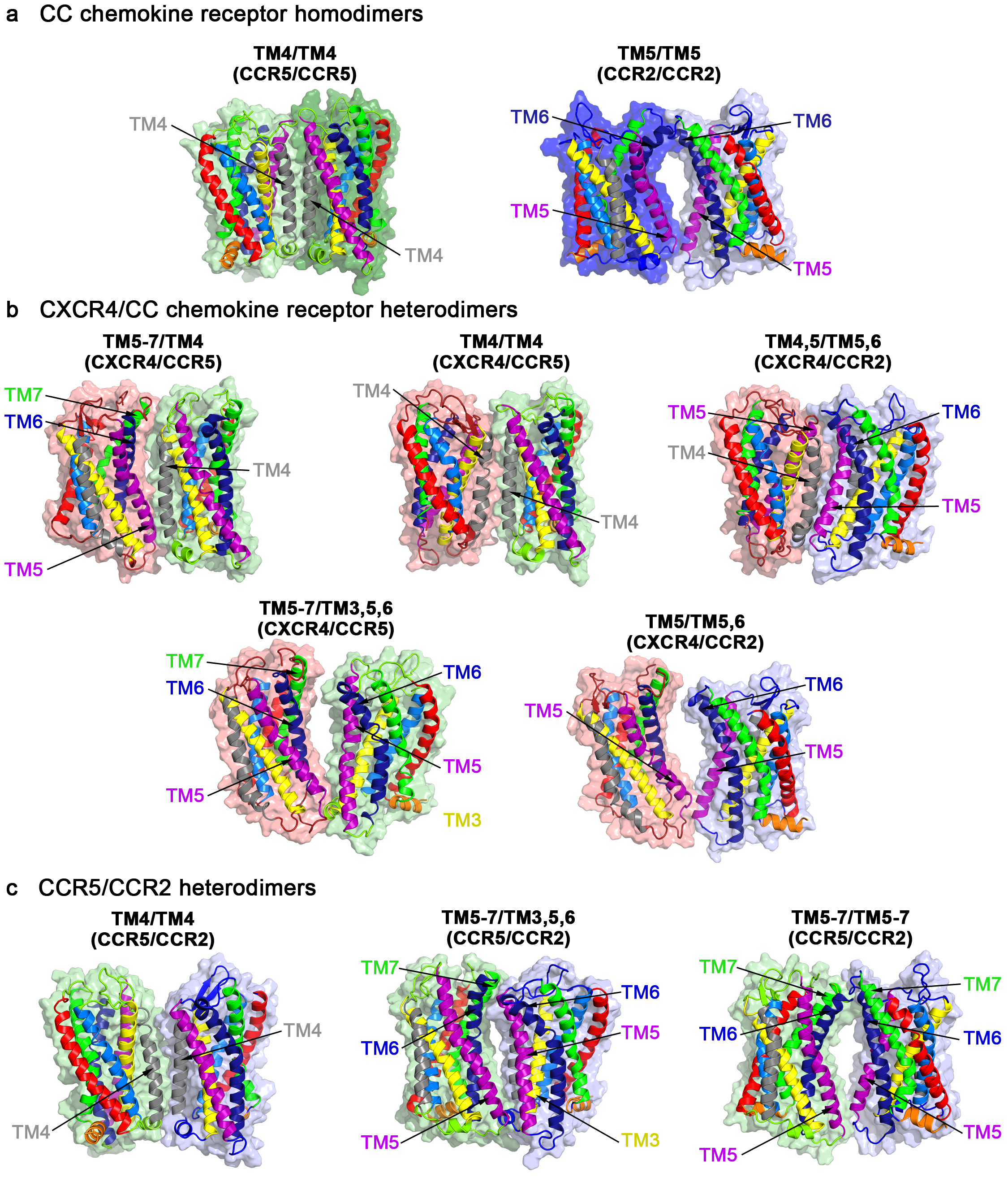

Supplement: S4 Fig — a CC chemokine receptor homodimers, b CXCR4/CC chemokine receptor heterodimers, and c CCR5/CCR2 heterodimers. The receptors are colored consistent with Fig 2. (TIF) [file pcbi.1006062.s004.tif]

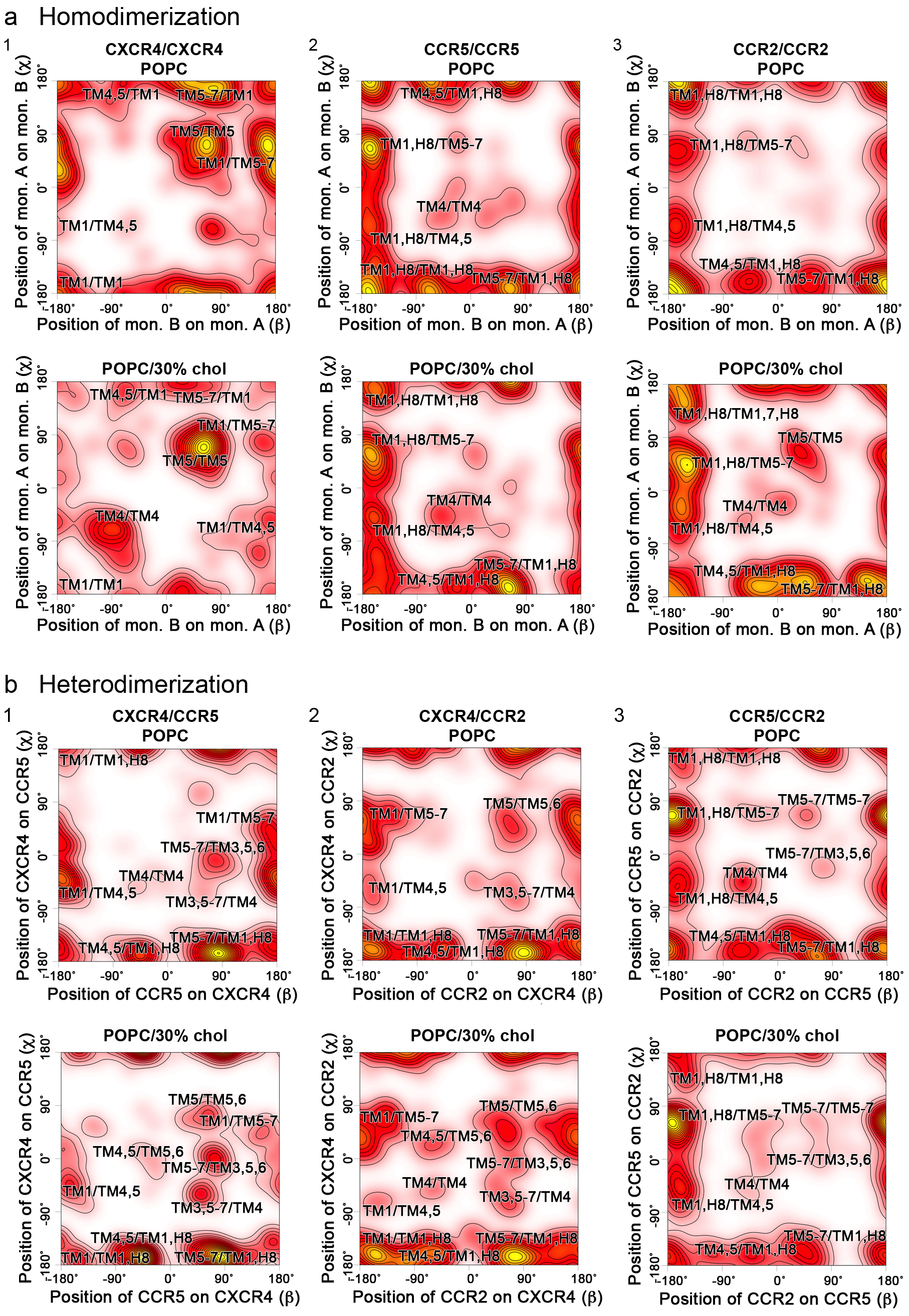

Supplement: S5 Fig — β denotes the binding position of monomer A on monomer B, whereas χ describes the angle under which monomer B binds to monomer A. Both angles were calculated for the last 50 ns for simulations in which dimers were formed. The most frequently observed (β, χ)-coordinates, i.e. dimer configurations, are labeled according to their corresponding dimer interfaces. a Densities of relative angles between monomers in chemokine receptor homodimers (a1: CXCR4 [47], a2: CCR5, a3: CCR2). b Densities of relative angles between monomers in chemokine receptor heterodimers (b1: CXCR4/CCR5, b2: CXCR4/CCR2, b3: CCR5/CCR2). (TIF) [file pcbi.1006062.s005.tif]

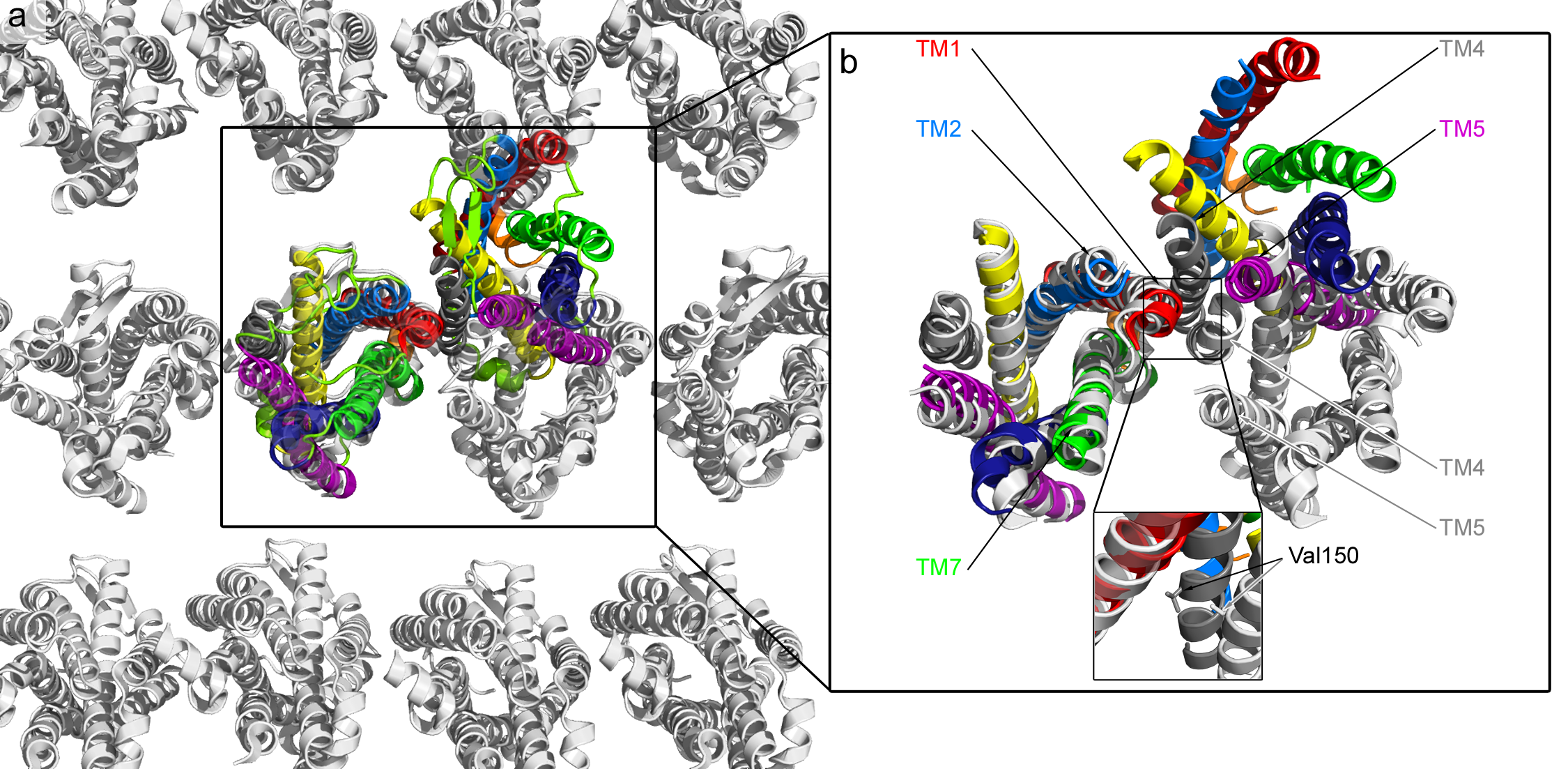

Supplement: S6 Fig — a Top view (extracellular site) onto the crystal packing observed for the CCR5 receptor TM1,7/TM4,5 dimer shown in grey (PDB: 4MBS [79]). The dimer structure obtained from simulations is colored according to Fig 2. The structural alignment was performed only on monomer A. b Enlarged view of the structural fit. Intra- and extracellular loops are not shown for clarity. (TIF) [file pcbi.1006062.s006.tif]
