# Supplementary material for: Closely related, yet unique: Distinct homo- and heterodimerization patterns of G protein coupled chemokine receptors and their fine-tuning by cholesterol
Source: PLoS Comput Biol. 2018 Mar 12;14(3):e1006062. doi: 10.1371/journal.pcbi.1006062 (PMC5864085; doi:10.1371/journal.pcbi.1006062)
Supplement: S1 Table — The lateral self-diffusion of proteins was calculated from the slope of the mean square displacement (MSD) averaged over the beads of each protein according to the Einstein relation: Ds=limt→∞⟨Δr(t)2⟩4t where Δr(t) denotes the distance a bead moved in time t. The MSD was calculated for the inital 200 ns of the simulations. In case of dimerization simulations, the MSD was calculated for simulations that did not show interaction energies between the monomers until 250 ns. The center of mass motion of the protein-membrane system was substracted during the MSD calculations. The slope of MSD curves was fitted on the time window between 5-20 ns. Data for CXCR4 was taken from Pluhackova et al. [47]. (PDF) [file pcbi.1006062.s007.pdf]

S1 Table Diffusion coefficients

System	**Diffusion coefficient** in 10^{-7} cm^2/s		
	**CXCR4**	**CCR5**	**CCR2**
**POPC (monomer)**	0.44±0.14	0.37±0.11	0.36±0.10
**POPC (homodimerization)**	0.51±0.13	0.48±0.13	0.47±0.13
**POPC/10% chol (monomer)**	0.33±0.03	0.25±0.05	0.29±0.09
**POPC/30% chol (homodimerization)**	0.26±0.06	0.23±0.06	0.23±0.06

Protein self-diffusion coefficients. According to the Einstein relation:

$$D_s = \lim_{t \to \infty} \frac{\langle \Delta r(t)^2 \rangle}{4t},$$

the lateral self-diffusion of proteins was calculated from the slope of the mean square displacement (MSD) averaged over all beads of each protein. Here, $\Delta r(t)$ denotes the distance a bead moved in time t. The MSD was calculated for the inital 200 ns of the simulations. In case of dimerization simulations, the MSD was calculated for simulations that did not show interaction energies between the monomers until 250 ns. For calculations of the MSD, the center of mass motion of the protein-membrane system was substracted. The slope of MSD curves was fitted on the time window between 5-20 ns. Data for CXCR4 was taken from Pluhackova *et al.*, PLoS Comput Biol, 2016;12(11):e1005169.
